# Supplementary material for: MolCryst-MLIPs: A Machine-Learned Interatomic Potentials Database for Molecular Crystals
Source: J Chem Theory Comput. 2026 Aug 27;22(17):8783–96. doi: 10.1021/acs.jctc.6c00735 (PMC13564353; doi:10.1021/acs.jctc.6c00735)
Supplement: Supplementary file 1 [file ct6c00735_si_001.pdf]

Supporting Information for:

MolCryst-MLIPs: A Machine-Learned Interatomic Potentials Database for Molecular Crystals

Adam Lahouari,[1,*] Shen Ai,[4] Jihye Han,[1] Jillian Hoffstadt,[1] Philipp Höllmer,[1,2,4] Charlotte Infante,[1] Pulkita Jain,[8] Sangram Kadam,[1] Maya M. Martirossyan,[1,4] Amara McCune,[4] Hypatia Newton,[2] Shlok J. Paul,[8] Willmor J. Peña Ccoa,[1] Jonathan Raghoonanan,[4,7] Sumon Sahu,[4] Oliver Tan,[1] Andrea Vergara,[4] Jutta Rogal,[3] and Mark E. Tuckerman[1,2,4,5,6]

[1]Department of Chemistry, New York University, New York, NY 10003, USA

[2]Simons Center for Computational Physical Chemistry, New York University, New York, NY 10003, USA

[3]Initiative for Computational Catalysis, Flatiron Institute, New York, NY 10010, USA

[4]Department of Physics, New York University, New York, NY 10003, USA

[5]Courant Institute of Mathematical Sciences, New York University, New York, NY 10012, USA

[6]NYU-ECNU Center for Computational Chemistry, Shanghai 200062, China

[7]New York University Shanghai, 567 West Yangsi Road, Pudong, Shanghai 200126, China

[8]Department of Chemical and Biomolecular Engineering, Tandon School of Engineering, New York University, Brooklyn, New York 11201, United States

[*] *Correspondence:* al9500@nyu.edu

A Computational Details

A.1 DFT Calculations

All *ab initio* calculations were performed using the Vienna Ab Initio Simulation Package (VASP) version 6.x with projector-augmented wave (PAW) pseudopotentials. The Perdew-Burke-Ernzerhof (PBE) exchange-correlation functional was employed in conjunction with the Grimme D4 dispersion correction to accurately capture van der Waals interactions critical for molecular crystal systems.

A.1.1 Geometry Optimization Parameters

DFT geometry optimizations (0 K) were performed with the following settings:

Table S1: DFT Cell Optimization Parameters

**Parameter**	**Value**
Exchange-correlation functional	PBE
Dispersion correction	Grimme D4 (IVDW = 13)
Plane-wave cutoff energy (ENCUT)	650 eV
Electronic convergence (EDIFF)	10^{-7} eV
Ionic relaxation algorithm (IBRION)	2 (Conjugate gradient)
Stress/cell optimization (ISIF)	3 (relax ions + cell shape + volume)
Maximum ionic steps (NSW)	150
Smearing method (ISMEAR)	−1 (Fermi smearing)
Smearing width (SIGMA)	0.05 eV
Precision (PREC)	Accurate
Real-space projection (LREAL)	Auto
Maximum electronic steps (NELM)	150

A.1.2 *Ab Initio* Molecular Dynamics Parameters

AIMD simulations were conducted in the canonical (NVT) ensemble using the Langevin thermostat with the following parameters:

Table S2: AIMD Simulation Parameters

**Parameter**	**Value**
Exchange-correlation functional	PBE
Dispersion correction	Grimme D4 (IVDW = 13)
Plane-wave cutoff energy (ENCUT)	650 eV
Electronic convergence (EDIFF)	10^{-7} eV
MD algorithm (IBRION)	0 (Molecular dynamics)
MD thermostat (MDALGO)	3 (Langevin)
Timestep (POTIM)	0.75 fs
Temperature range	25–500 K
Friction coefficients (LANGEVIN_GAMMA)	10.0 ps^{-1} (all species)
Lattice friction (LANGEVIN_GAMMA_L)	1.0
Total MD steps (NSW)	50,000
Stress/cell control (ISIF)	2 (relax ions only)
Smearing method (ISMEAR)	-1 (Fermi smearing)
Smearing width (SIGMA)	0.05 eV
Precision (PREC)	High
Real-space projection (LREAL)	Auto
Symmetry (ISYM)	0 (off for MD)
Output frequency (NBLOCK)	1
k-point blocking (KBLOCK)	10

Table S3: DFT-Optimized and Experimental Structures for All Polymorphs

**Compound**	**Polymorph**	**Type**	$\Delta E^{relative}_{\textbf{lattice}}$ **(kJ/mol)**	$E_{\textbf{per mol}}$ **(eV)**	a **(Å)**	b **(Å)**	c **(Å)**	α **(°)**	β **(°)**	γ **(°)**	**Volume** **(Å^3)**	ΔV **(%)**
Resorcinol [46, 47]	resora03	DFT-Opt	1.55	−90.946	10.333	9.266	5.562	90.00	90.00	90.00	532.55	−4.6
		Exp	—	—	10.470	9.406	5.666	90.00	90.00	90.00	557.95	—
	resora08	DFT-Opt	4.64	−90.914	7.785	12.467	5.360	90.00	90.00	90.00	520.26	−5.6
		Exp	—	—	7.934	12.606	5.511	90.00	90.00	90.00	551.19	—
	resora13	DFT-Opt	0.00	−90.962	10.339	9.289	5.555	90.00	90.00	90.00	533.52	−5.1
		Exp	—	—	10.530	9.530	5.600	90.00	90.00	90.00	561.97	—
	resora16	DFT-Opt	1.27	−90.948	10.327	9.250	5.574	90.00	90.00	90.00	532.48	−1.0
		Exp	—	—	10.344	9.271	5.611	90.00	90.00	90.00	538.10	—
Pyrazinamide [31, 48–51]	pyrzin01	DFT-Opt	0.75	−96.262	14.182	3.620	10.476	90.00	100.76	90.00	528.41	−5.6
		Exp	—	—	14.372	3.711	10.726	90.00	101.92	90.00	559.73	—
	pyrzin02	DFT-Opt	3.11	−96.237	5.655	5.118	9.764	98.08	97.62	106.94	263.13	−5.9
		Exp	—	—	5.728	5.221	9.945	96.81	97.27	106.22	279.56	—
	pyrzin14	DFT-Opt	3.31	−96.235	22.647	6.704	3.595	90.00	100.82	90.00	536.12	−7.2
		Exp	—	—	23.200	6.770	3.750	90.00	101.20	90.00	577.77	—
	pyrzin15	DFT-Opt	3.40	−96.234	3.596	6.701	22.246	90.00	92.32	90.00	535.72	−2.0
		Exp	—	—	3.615	6.738	22.464	90.00	92.50	90.00	546.64	—
	pyrzin16	DFT-Opt	2.47	−96.244	5.112	5.655	9.780	97.61	98.03	106.97	263.24	−2.1
		Exp	—	—	5.119	5.705	9.857	97.46	98.17	106.47	268.82	—

Continued on next page

Table S3 – *Continued from previous page*

**Compound**	**Polymorph**	**Type**	$\Delta E_{\mathbf{lattice}}^{relative}$ **(kJ/mol)**	$E_{\mathbf{per\ mol}}$ **(eV)**	a **(Å)**	b **(Å)**	c **(Å)**	α **(°)**	β **(°)**	γ **(°)**	**Volume (Å^3)**	ΔV **(%)**
	pyrzin17	DFT-Opt	4.32	−96.225	7.124	3.664	10.457	90.00	106.00	90.00	262.37	−4.9
		Exp	—	—	7.183	3.729	10.758	90.00	106.77	90.00	275.92	—
	pyrzin18	DFT-Opt	0.81	−96.261	14.188	3.618	10.473	90.00	100.72	90.00	528.17	−2.3
		Exp	—	—	14.315	3.624	10.616	90.00	101.12	90.00	540.35	—
	pyrzin20	DFT-Opt	4.35	−96.225	7.124	3.663	10.458	90.00	106.02	90.00	262.34	−1.8
		Exp	—	—	7.170	3.648	10.648	90.00	106.35	90.00	267.23	—
	pyrzin21	DFT-Opt	3.37	−96.235	3.597	6.701	22.248	90.00	91.95	90.00	535.97	−3.3
		Exp	—	—	3.654	6.736	22.537	90.00	92.22	90.00	554.29	—
	pyrzin22	DFT-Opt	3.40	−96.234	3.596	6.701	22.252	90.00	92.47	90.00	535.75	−2.1
		Exp	—	—	3.617	6.741	22.462	90.00	92.39	90.00	547.28	—
	pyrzin23	DFT-Opt	0.00	−96.269	14.190	3.619	10.468	90.00	100.69	90.00	528.20	−2.3
		Exp	—	—	14.340	3.621	10.613	90.00	101.04	90.00	540.88	—
	pyrzin25	DFT-Opt	3.16	−96.237	5.111	5.654	9.786	97.63	98.01	107.04	263.24	−1.5
		Exp	—	—	5.103	5.708	9.849	97.53	98.50	106.56	267.37	—
	pyrzin27	DFT-Opt	3.42	−96.234	3.596	6.701	22.251	90.00	92.67	90.00	535.58	−1.5
		Exp	—	—	3.588	6.761	22.434	90.00	92.75	90.00	543.62	—
	pyrzin29	DFT-Opt	0.82	−96.261	14.193	3.619	10.464	90.00	100.67	90.00	528.13	−2.0
		Exp	—	—	14.343	3.609	10.602	90.00	100.98	90.00	538.72	—

Continued on next page

Table S3 – *Continued from previous page*

**Compound**	**Polymorph**	**Type**	$\Delta E^{relative}_{\mathbf{lattice}}$ **(kJ/mol)**	$E_{\mathbf{per\ mol}}$ **(eV)**	a **(Å)**	b **(Å)**	c **(Å)**	α **(°)**	β **(°)**	γ **(°)**	**Volume (Å^3)**	ΔV **(%)**
Niacinamide [29, 52, 53]	nicoam01	DFT-Opt	0.03	−101.085	3.856	15.472	9.226	90.00	97.38	90.00	545.80	−2.7
		Exp	—	—	3.877	15.600	9.375	90.00	98.45	90.00	560.86	—
	nicoam05	DFT-Opt	0.00	−101.086	3.859	15.444	9.228	90.00	97.32	90.00	545.47	−5.8
		Exp	—	—	3.974	15.642	9.430	90.00	99.02	90.00	578.98	—
	nicoam07	DFT-Opt	1.47	−101.070	3.770	14.287	5.076	90.00	94.21	90.00	272.63	−2.6
		Exp	—	—	3.812	14.388	5.119	90.00	94.26	90.00	280.03	—
	nicoam08	DFT-Opt	0.99	−101.075	3.724	12.274	12.960	71.11	84.77	84.54	556.74	−2.3
		Exp	—	—	3.752	12.323	13.062	71.50	85.68	85.20	570.01	—
	nicoam13	DFT-Opt	0.03	−101.085	3.857	15.481	9.219	90.00	97.31	90.00	545.97	−3.2
		Exp	—	—	3.883	15.645	9.384	90.00	98.39	90.00	563.90	—
	nicoam17	DFT-Opt	2.47	−101.060	10.077	5.960	9.736	90.00	100.00	90.00	575.83	−2.2
		Exp	—	—	9.901	5.877	10.278	90.00	100.00	90.00	589.03	—
	nicoam18	DFT-Opt	3.02	−101.054	7.429	7.905	10.694	108.02	102.72	96.55	571.24	−2.4
		Exp	—	—	7.556	7.941	10.797	108.10	102.60	98.29	585.14	—
Nicotinic acid [54–56]	nicoac01	DFT-Opt	0.00	−95.781	7.128	11.585	7.001	90.00	114.54	90.00	525.96	−5.7
		Exp	—	—	7.162	11.703	7.242	90.00	113.20	90.00	557.92	—
	nicoac05	DFT-Opt	1.63	−95.764	7.134	11.597	6.998	90.00	114.97	90.00	524.85	−8.4
		Exp	—	—	7.303	11.693	7.330	90.00	113.68	90.00	573.24	—

Continued on next page

Table S3 – *Continued from previous page*

**Compound**	**Polymorph**	**Type**	$\Delta E^{relative}_{\textbf{lattice}}$ **(kJ/mol)**	$E_{\textbf{per mol}}$ **(eV)**	a **(Å)**	b **(Å)**	c **(Å)**	α **(°)**	β **(°)**	γ **(°)**	**Volume (Å^3)**	ΔV **(%)**
	nicoac07	DFT-Opt	0.89	−95.772	7.141	11.593	7.002	90.00	115.13	90.00	524.70	−2.8
		Exp	—	—	7.167	11.671	7.106	90.00	114.78	90.00	539.63	—
	nicoac08	DFT-Opt	1.70	−95.763	7.129	11.589	6.995	90.00	114.77	90.00	524.67	−5.5
		Exp	—	—	7.176	11.670	7.227	90.00	113.52	90.00	554.93	—
Isonicotinamide [28, 57–59]	ehowih	DFT-Opt	1.40	−101.057	10.088	5.692	9.899	90.00	98.00	90.00	562.92	−2.7
		Exp	—	—	10.166	5.730	10.033	90.00	98.17	90.00	578.55	—
	ehowih01	DFT-Opt	1.41	−101.057	10.098	5.687	9.900	90.00	98.02	90.00	563.01	−2.8
		Exp	—	—	10.176	5.732	10.034	90.00	98.04	90.00	579.48	—
	ehowih03	DFT-Opt	0.69	−101.065	10.002	7.228	15.689	90.00	90.00	90.00	1134.33	−3.9
		Exp	—	—	10.160	7.323	15.872	90.00	90.00	90.00	1180.95	—
	ehowih05	DFT-Opt	0.00	−101.072	5.053	9.286	12.019	90.00	89.67	90.00	563.97	−6.4
		Exp	—	—	5.192	9.466	12.259	90.00	91.22	90.00	602.40	—
	ehowih07	DFT-Opt	1.38	−101.058	10.088	5.693	9.900	90.00	97.92	90.00	563.10	−4.5
		Exp	—	—	10.229	5.754	10.095	90.00	97.28	90.00	589.36	—
Durene [23]	durene01	DFT-Opt	0.00	−144.014	11.583	5.493	6.753	90.00	112.56	90.00	396.82	−8.2
		Exp	—	—	11.570	5.770	7.030	90.00	112.93	90.00	432.23	—
	durene05	DFT-Opt	0.05	−144.013	6.759	11.581	5.492	90.00	90.00	112.71	396.55	−7.6
		Exp	—	—	7.023	11.570	5.733	90.00	90.00	112.87	429.21	—

Continued on next page

Table S3 – *Continued from previous page*

**Compound**	**Polymorph**	**Type**	$\Delta E^{relative}_{\mathbf{lattice}}$ **(kJ/mol)**	$E_{\mathbf{per\ mol}}$ **(eV)**	a **(Å)**	b **(Å)**	c **(Å)**	α **(°)**	β **(°)**	γ **(°)**	**Volume (Å^3)**	ΔV **(%)**
	durene06	DFT-Opt	0.09	−144.013	6.770	5.493	10.912	90.00	102.10	90.00	396.75	−3.4
		Exp	—	—	6.890	5.620	10.873	90.00	102.72	90.00	410.69	—
	coumar01	DFT-Opt	1.42	−117.840	15.277	5.491	7.824	90.00	90.00	90.00	656.31	−5.5
		Exp	—	—	15.500	5.664	7.915	90.00	90.00	90.00	694.87	—
	coumar02	DFT-Opt	1.45	−117.841	15.277	5.491	7.822	90.00	90.00	90.00	656.17	−5.7
		Exp	—	—	15.503	5.666	7.918	90.00	90.00	90.00	695.52	—
	coumar11	DFT-Opt	1.57	−117.839	5.489	7.832	15.265	90.00	90.00	90.00	656.14	−2.3
		Exp	—	—	5.609	7.734	15.478	90.00	90.00	90.00	671.47	—
	coumar12	DFT-Opt	1.51	−117.840	15.259	5.492	7.830	90.00	90.00	90.00	656.15	−5.5
		Exp	—	—	15.502	5.663	7.910	90.00	90.00	90.00	694.43	—
	coumar13	DFT-Opt	0.00	−117.856	3.761	15.360	5.749	90.00	94.82	90.00	330.93	−6.9
		Exp	—	—	3.980	15.291	5.858	90.00	94.24	90.00	355.50	—
Coumarin [22, 60]	coumar14	DFT-Opt	1.38	−117.841	16.759	5.860	13.652	90.00	90.00	90.00	1340.69	−2.6
		Exp	—	—	16.782	5.921	13.852	90.00	90.00	90.00	1376.56	—
	coumar18	DFT-Opt	1.93	−117.836	4.757	6.792	20.335	90.00	90.00	90.00	657.05	−5.9
		Exp	—	—	4.868	6.882	20.851	90.00	90.00	90.00	698.47	—
	coumar19	DFT-Opt	1.54	−117.840	15.268	5.493	7.825	90.00	90.00	90.00	656.24	−4.0
		Exp	—	—	15.500	5.636	7.822	90.00	90.00	90.00	683.35	—

Continued on next page

Table S3 – *Continued from previous page*

**Compound**	**Polymorph**	**Type**	$\Delta E^{relative}_{\textbf{lattice}}$ **(kJ/mol)**	$E_{\textbf{per mol}}$ **(eV)**	a **(Å)**	b **(Å)**	c **(Å)**	α **(°)**	β **(°)**	γ **(°)**	**Volume (Å^3)**	ΔV **(%)**
	coumar20	DFT-Opt	0.12	−117.854	3.759	15.312	5.766	90.00	94.17	90.00	331.04	−3.7
		Exp	—	—	3.870	15.284	5.821	90.00	93.61	90.00	343.63	—
	coumar21	DFT-Opt	1.54	−117.840	15.290	5.495	7.812	90.00	90.00	90.00	656.44	−5.7
		Exp	—	—	15.526	5.669	7.912	90.00	90.00	90.00	696.37	—
	coumar22	DFT-Opt	1.54	−117.840	15.283	5.491	7.821	90.00	90.00	90.00	656.37	−5.7
		Exp	—	—	15.521	5.663	7.916	90.00	90.00	90.00	695.78	—
	coumar23	DFT-Opt	1.51	−117.840	15.278	5.491	7.822	90.00	90.00	90.00	656.19	−3.2
		Exp	—	—	15.496	5.617	7.785	90.00	90.00	90.00	677.69	—
Benzoic acid [61, 62]	benzac01	DFT-Opt	0.94	−100.387	5.346	4.989	21.604	90.00	98.61	90.00	569.73	−8.0
		Exp	—	—	5.510	5.157	21.973	90.00	97.41	90.00	619.15	—
	benzac02	DFT-Opt	1.04	−100.386	5.381	5.003	21.426	90.00	98.69	90.00	570.11	−7.1
		Exp	—	—	5.500	5.128	21.950	90.00	97.37	90.00	613.96	—
	benzac12	DFT-Opt	0.00	−100.396	5.348	4.985	21.466	90.00	95.63	90.00	569.56	−2.9
		Exp	—	—	5.415	5.039	21.630	90.00	96.14	90.00	586.82	—
	benzac20	DFT-Opt	0.92	−100.387	5.345	4.989	21.476	90.00	95.64	90.00	569.96	−4.0
		Exp	—	—	5.438	5.060	21.706	90.00	96.18	90.00	593.74	—
	benzac22	DFT-Opt	1.20	−100.383	5.391	4.996	21.289	90.00	95.85	90.00	570.49	−7.2
		Exp	—	—	5.502	5.137	21.927	90.00	97.05	90.00	615.06	—

Continued on next page

Table S3 – *Continued from previous page*

**Compound**	**Polymorph**	**Type**	$\Delta E^{relative}_{\mathbf{lattice}}$ **(kJ/mol)**	$E_{\mathbf{per\ mol}}$ **(eV)**	a **(Å)**	b **(Å)**	c **(Å)**	α **(°)**	β **(°)**	γ **(°)**	**Volume (Å^3)**	ΔV **(%)**
	benzac23	DFT-Opt	1.10	−100.385	4.992	5.367	21.371	95.72	89.98	89.99	569.79	−8.5
		Exp	—	—	5.156	5.521	22.049	97.19	90.00	90.00	622.72	—
Benzamide [63–66]	bzamid01	DFT-Opt	0.30	−104.609	5.496	4.991	21.167	90.00	88.50	90.00	580.42	−7.0
		Exp	—	—	5.607	5.046	22.053	90.00	90.66	90.00	623.90	—
	bzamid07	DFT-Opt	1.38	−104.598	5.551	5.072	20.933	90.00	88.75	90.00	589.19	−5.8
		Exp	—	—	5.609	5.040	22.117	90.00	90.64	90.00	625.23	—
	bzamid08	DFT-Opt	0.11	−104.611	5.012	5.362	22.172	90.00	102.36	90.00	581.92	−7.3
		Exp	—	—	5.055	5.514	22.956	90.00	101.29	90.00	627.50	—
	bzamid11	DFT-Opt	0.08	−104.612	5.010	5.363	21.669	90.00	90.72	90.00	582.27	−3.2
		Exp	—	—	5.052	5.424	21.949	90.00	90.91	90.00	601.41	—
	bzamid12	DFT-Opt	0.02	−104.612	5.010	5.366	22.295	90.00	103.71	90.00	582.31	−4.9
		Exp	—	—	5.059	5.461	22.833	90.00	103.85	90.00	612.46	—
	bzamid14	DFT-Opt	0.40	−104.608	5.498	4.992	21.145	90.00	91.52	90.00	580.16	−4.8
		Exp	—	—	5.574	5.036	21.702	90.00	90.50	90.00	609.20	—
	bzamid15	DFT-Opt	0.34	−104.609	5.495	4.990	21.175	90.00	91.49	90.00	580.35	−4.8
		Exp	—	—	5.567	5.032	21.754	90.00	90.18	90.00	609.32	—
	bzamid16	DFT-Opt	0.00	−104.612	5.010	5.364	22.310	90.00	103.72	90.00	582.42	−4.1
		Exp	—	—	5.048	5.447	22.759	90.00	103.86	90.00	607.54	—

Continued on next page

Table S3 – *Continued from previous page*

**Compound**	**Polymorph**	**Type**	$\Delta E^{relative}_{\mathbf{lattice}}$ **(kJ/mol)**	$E_{\mathbf{per\ mol}}$ **(eV)**	a **(Å)**	b **(Å)**	c **(Å)**	α **(°)**	β **(°)**	γ **(°)**	**Volume (Å^3)**	ΔV **(%)**
	bzamid17	DFT-Opt	0.16	−104.611	5.011	5.364	22.284	90.00	103.71	90.00	581.93	−3.3
		Exp	—	—	5.042	5.430	22.639	90.00	103.80	90.00	601.95	—
	bzamid18	DFT-Opt	0.36	−104.609	5.492	4.989	21.185	90.00	91.48	90.00	580.27	−4.7
		Exp	—	—	5.560	5.040	21.720	90.00	90.10	90.00	608.65	—

A.2 Dataset Energy-Force Distributions

Figure S1 shows 2D density plots of energy per atom versus mean atomic force magnitude for each compound. All datasets exhibit strong positive correlations ($r > 0.9$), consistent with the expected physical relationship between configuration energy and atomic forces, confirming the internal consistency of the DFT reference data across both near- and off-equilibrium regions.

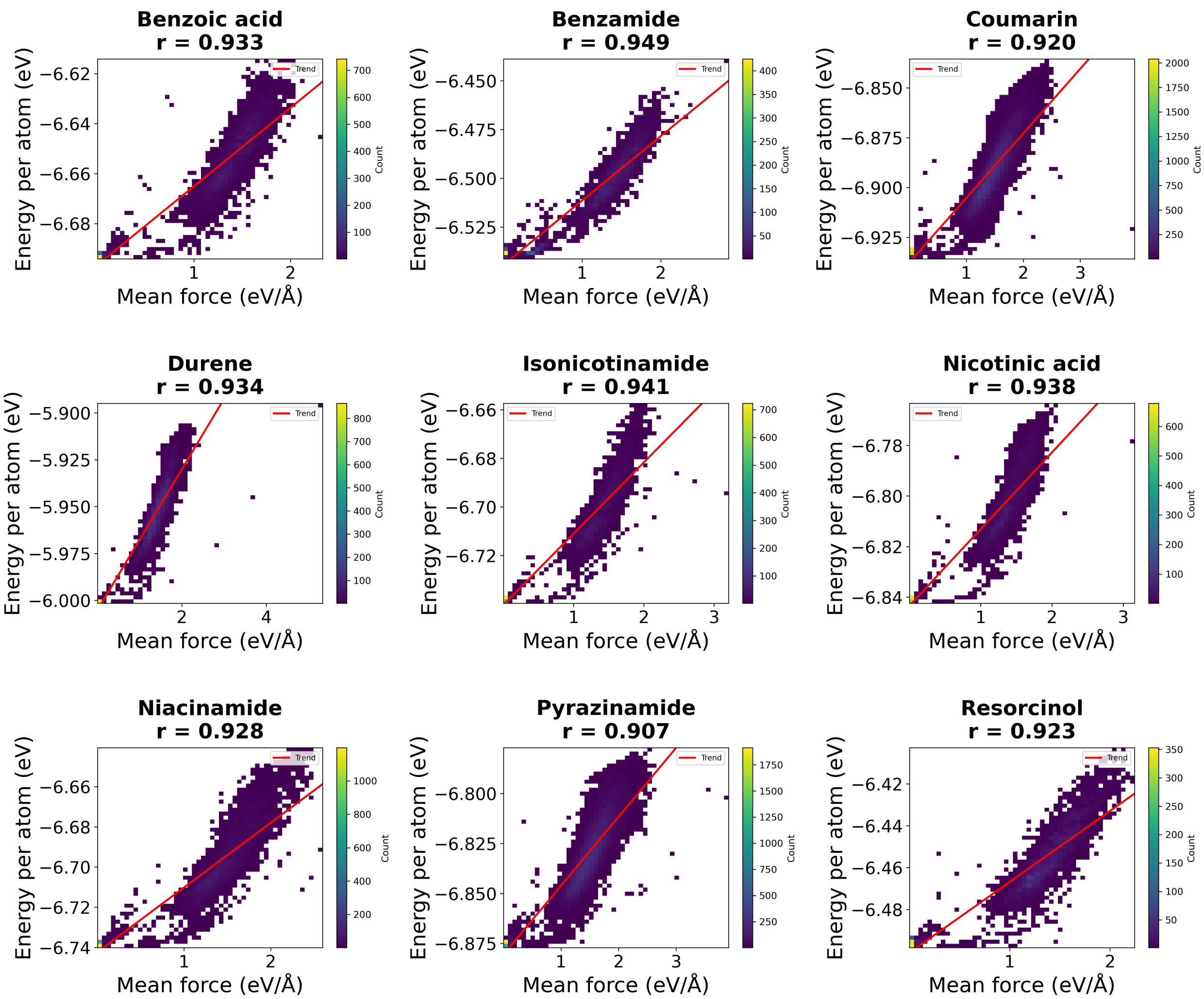

Figure S1: Energy-force density distributions for all nine molecular crystal datasets. Each panel shows the 2D histogram of energy per atom versus mean atomic force magnitude, with color indicating configuration count. Red lines show linear fits, and Pearson correlation coefficients (r) are indicated.

A.3 MACE Model Training

A.3.1 Foundation Model and Architecture

A.3.2 Training Hyperparameters

Table S4: MACE fine-tuning hyperparameters used for all nine systems.

**Parameter**	**Value**
Architecture	
Foundation model	MACE-MH-1 (`omol` head)
Hidden irreducible representations	$512 \times 0e + 512 \times 1o$
Correlation order	3 (body order: 4)
Spherical harmonics up to	$\ell = 3$
Radial cutoff ($r_{\max}$)	6.0 Å
Total receptive field	12.0 Å
Number of interaction layers	2
Radial basis transform	Agnesi
Training Protocol	
Batch size	10
Optimizer	Adam
Initial learning rate	1×10^{-3}
Weight decay	5×10^{-7}
Exponential moving average (EMA) decay	0.99
Loss Function Weights (Epochs 0–100)	
Energy weight	100.0
Forces weight	100.0
Stage Two (Epochs 100–300)	
Stage two learning rate	1×10^{-4}
Energy weight	100.0
Forces weight	100.0
Data Processing	
Train/validation split	85/15
Data format	HDF5

A.3.3 Isolated Atom Energies

The following isolated atom reference energies (E0s) were used for all systems containing H, C, N, and O. Each was computed with VASP for a single atom in a large cubic box, using the PBE functional (ENCUT = 850 eV, Γ-point only, `PREC = High`, EDIFF = 10^{-6} eV), with spin polarization enabled for open-shell atoms (C, N, O) following Hund's first rule.

Table S5: Isolated Atom Reference Energies

**Element**	**Energy (eV)**
H (Z=1)	−0.00292689
C (Z=6)	−1.24732389
N (Z=7)	−3.12548797
O (Z=8)	−1.5314622

A.4 Correlation Plots and training time

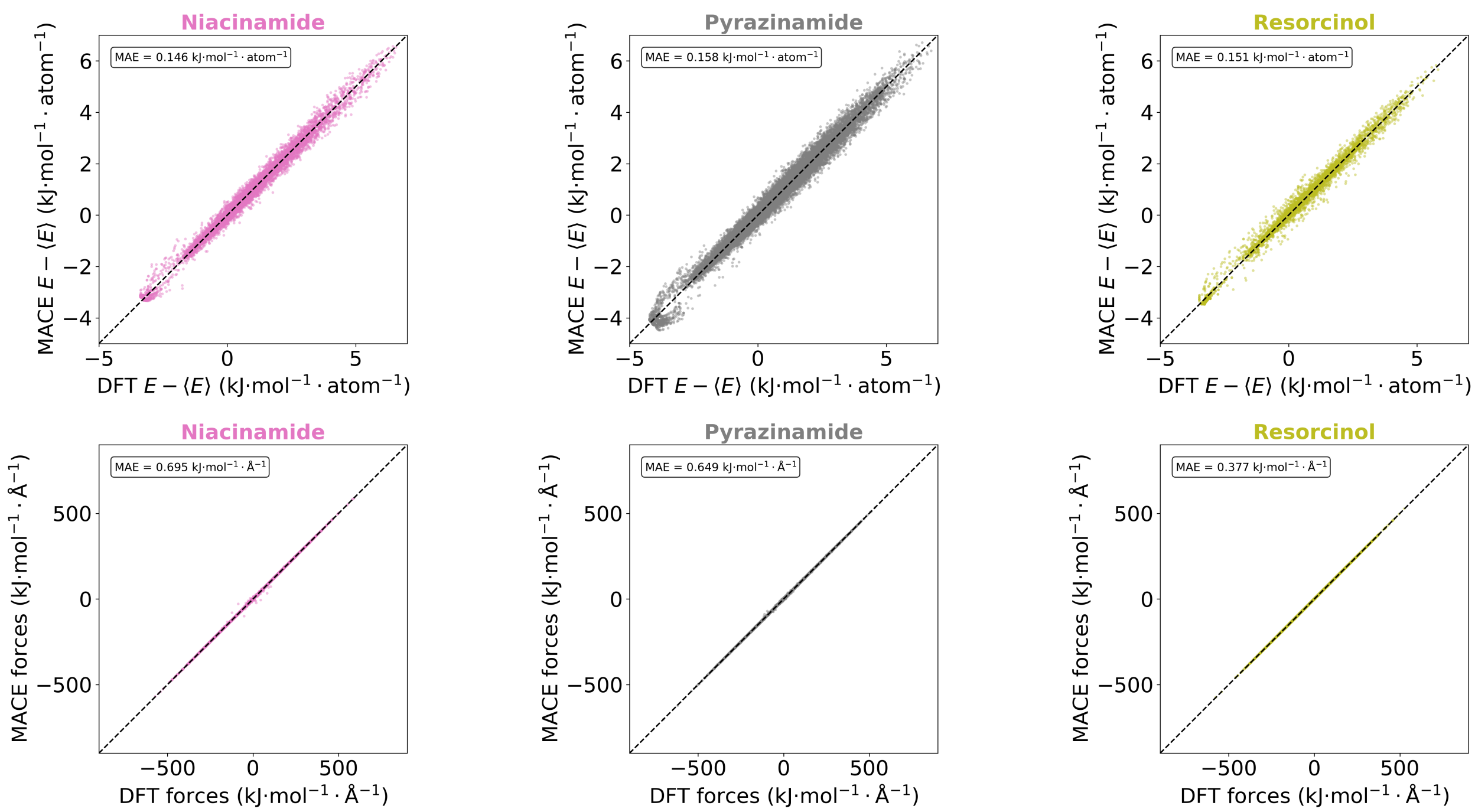

Figure S2: Force and energy correlation plots for the second set of fine-tuned MACE models (EHOWIH, NICOAC, NICOAM, PYRIZIN). Each panel shows MACE-predicted values against DFT reference values. The dashed line indicates perfect agreement.

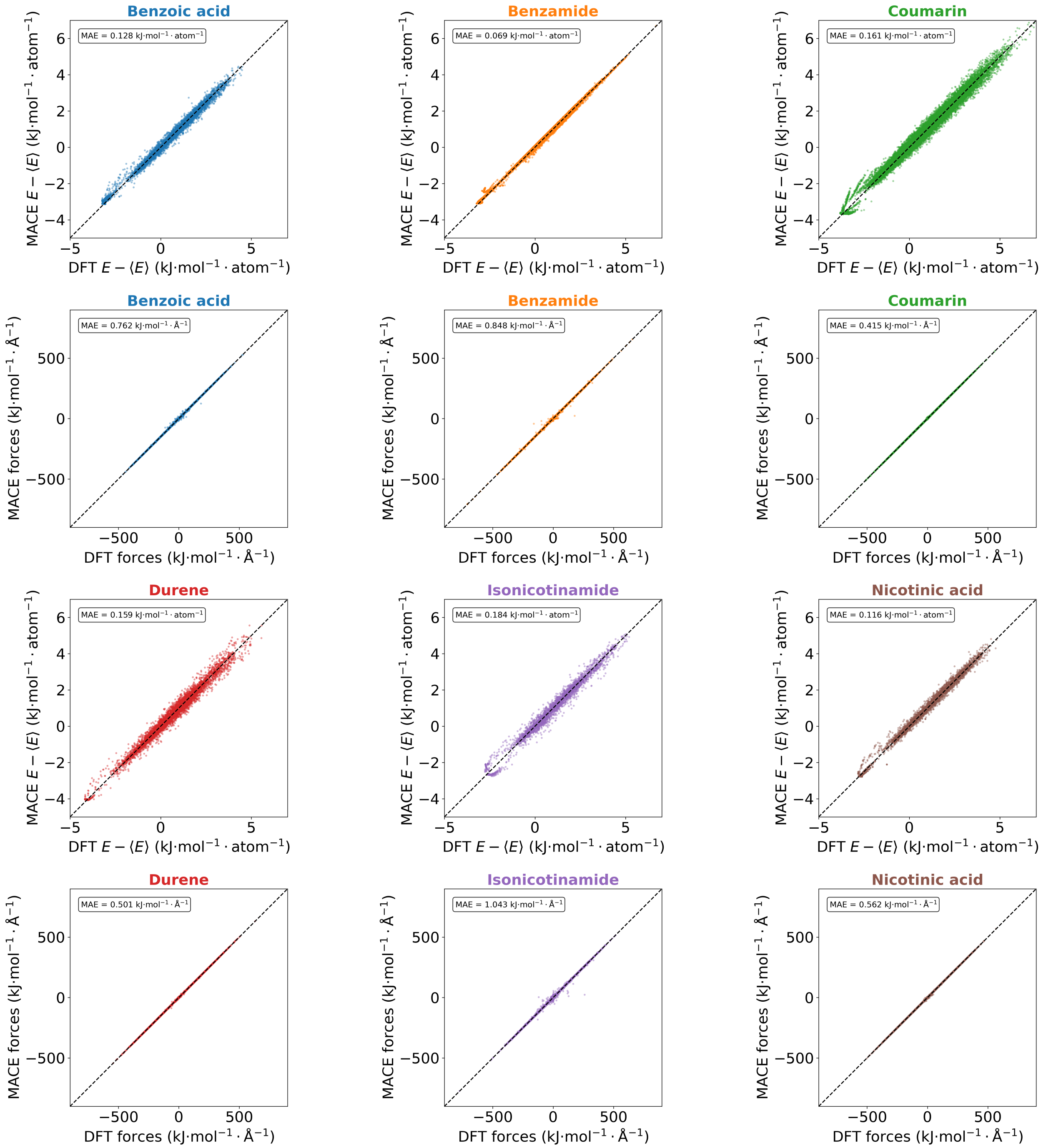

Figure S3: Force and energy correlation plots for the first set of fine-tuned MACE models (BENZAC, BZAMID, COUMAR, DURENE). Each panel shows MACE-predicted values against DFT reference values. The dashed line indicates perfect agreement.

Table S6: Training dataset sizes and approximate wall-clock training times for all nine MC-MLIP models. Training times are estimated from MACE training log timestamps. All models were trained on a single NVIDIA A100 GPU.

**Compound**	**Training configs**	**Total time (h)**
Benzoic acid	6,195	∼34
Benzamide	8,880	∼55
Coumarin	23,985	∼115
Durene	6,045	∼28
Isonicotinamide	3,465	∼25
Nicotinic acid	4,395	∼22
Niacinamide	8,250	∼35
Pyrazinamide	25,845	∼107
Resorcinol	4,035	∼22

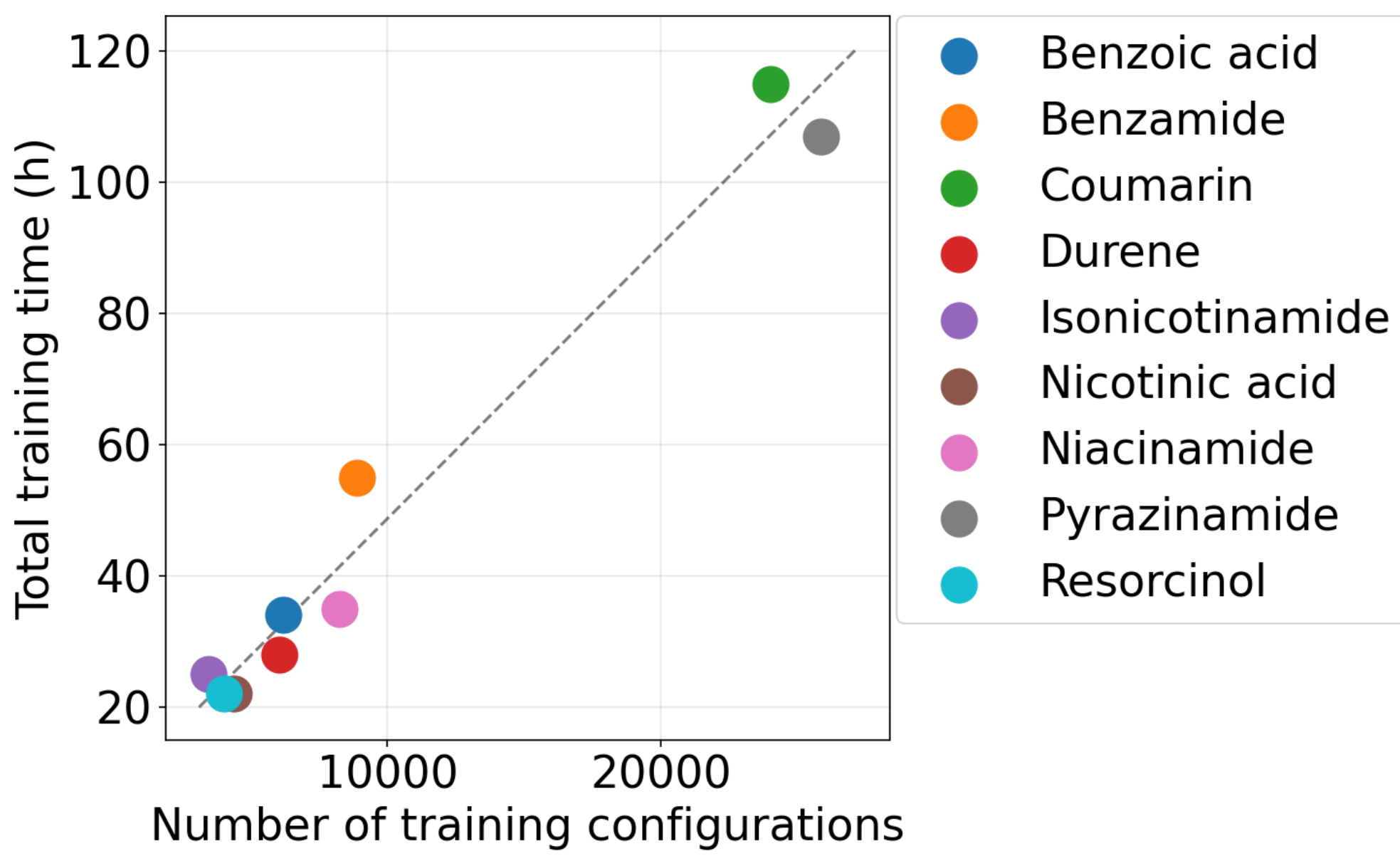

Figure S4: Number of training configurations versus total wall-clock training time for the nine MC-MLIP models. The dashed line shows a linear fit to the data. All models were trained on a single NVIDIA A100 GPU.

B DFT-Optimized Structures for All Polymorphs

This section provides comprehensive structural data for all polymorphs studied in this work. For each polymorph, we present both the DFT-optimized structure (at 0 K) and the experimental structure (typically measured near 298 K) for comparison.

B.1 MACE-Optimized Polymorph Energetics

Table S7: Correspondence between Roman numeral labels used in Figure 2 and CSD reference codes for each polymorph. Polymorphs are ordered by increasing DFT relative lattice energy (ΔE_{latt}).

**Coumarin**		**Isonicotinamide**		**Niacinamide**		**Pyrazinamide**	
Label	CSD Code	Label	CSD Code	Label	CSD Code	Label	CSD Code
I	COUMAR20	I	EHOWIH05	I	NICOAM01	I	PYRZIN01
II	COUMAR13	II	EHOWIH07	II	NICOAM13	II	PYRZIN14
III	COUMAR21	III	EHOWIH01	III	NICOAM05	III	PYRZIN23
IV	COUMAR22	IV	EHOWIH	IV	NICOAM08	IV	PYRZIN18
V	COUMAR12			V	NICOAM07	V	PYRZIN29
VI	COUMAR01			VI	NICOAM18	VI	PYRZIN02
VII	COUMAR02			VII	NICOAM17	VII	PYRZIN16
VIII	COUMAR19					VIII	PYRZIN25
IX	COUMAR23					IX	PYRZIN21
X	COUMAR11					X	PYRZIN22
XI	COUMAR18					XI	PYRZIN15
						XII	PYRZIN27

To validate the transferability of the fine-tuned MACE models beyond the training data, we performed geometry optimizations on all experimental polymorphs from the Cambridge Structural Database using the trained potentials. Figure S5 shows the relative lattice energy (ΔE_{latt}) versus density for each system, where energies are referenced to the most stable polymorph (set to zero).

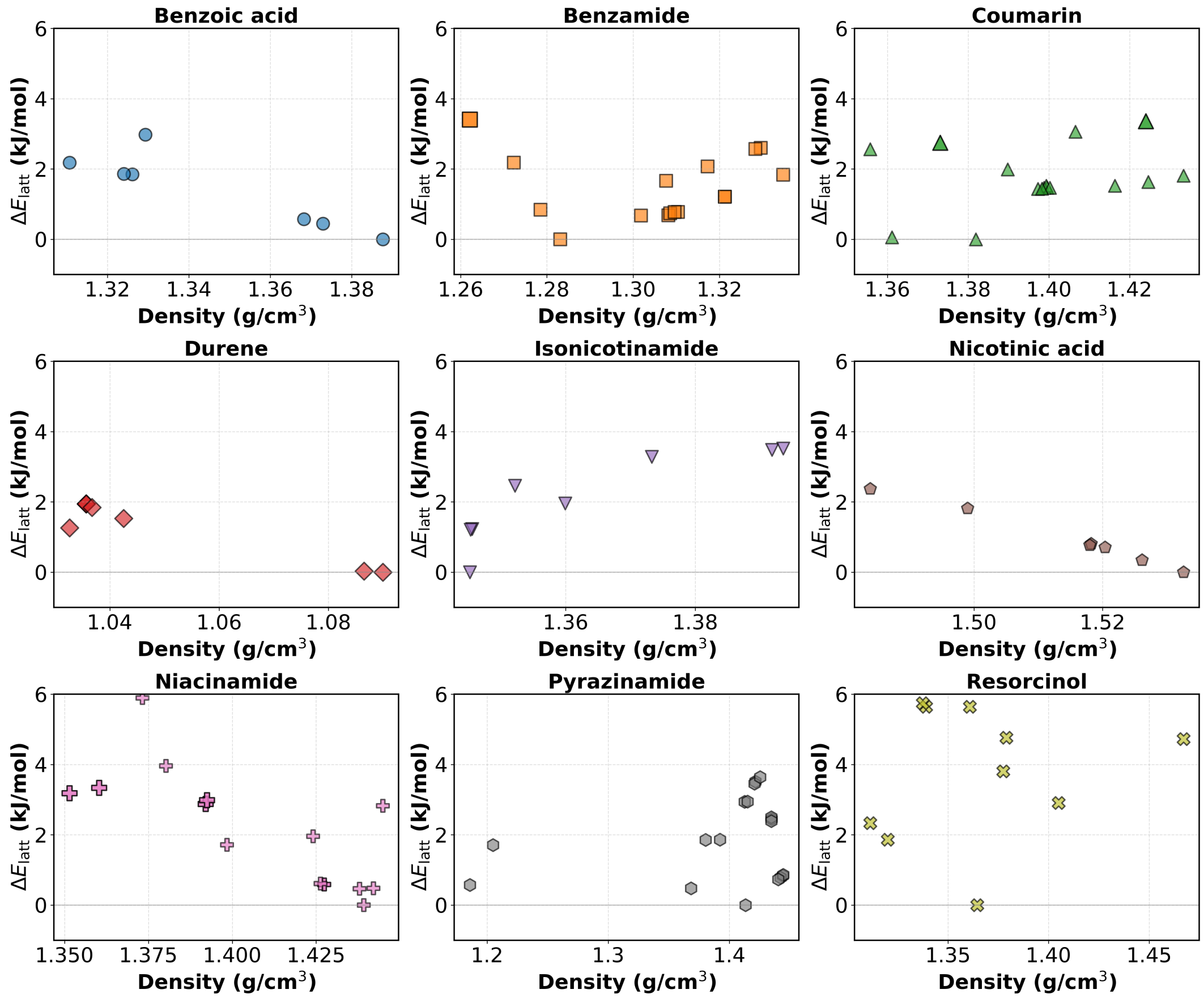

Figure S5: Relative lattice energy (ΔE_{latt}) versus density for MACE-optimized polymorphs. Each point represents a distinct polymorph, with the most stable structure (lowest energy) set as the reference ($\Delta E = 0$).

Table S8: Per-system accuracy of the relative lattice energy ΔE_{latt} against the PBE+D4 reference, referenced to the DFT-most-stable polymorph. Spread: range of ΔE_{latt} predicted by each method (kJ mol^{-1}), obtained by relaxing the DFT-optimized structures with that method's own potential; a model-level quantity.

; MAE and RMSE in kJ mol^{-1} per molecule; τ, ρ, and r: Kendall, Spearman, and Pearson correlations with the DFT energies/ordering; penalty: ΔE_{latt} of the predicted most stable polymorph above the DFT minimum (kJ mol^{-1}); min: most stable polymorph correctly identified.

System	Model	spread	MAE	RMSE	$\mathrm{MAE}_{\mathrm{pair}}$	τ	ρ	r	Penalty	min
Benzoic acid	**MolCryst**	2.53	1.29	1.57	1.26	0.33	0.40	0.34	0.00	✓
	`omol`	2.53	1.62	1.88	1.27	−0.80	−0.90	−0.85	2.53	×
	Orb	2.53	1.60	1.89	1.37	−0.40	−0.50	−0.65	2.53	×
	UMA	2.53	1.29	1.56	1.20	−0.40	−0.40	−0.21	2.53	×
Benzamide	**MolCryst**	2.60	0.79	0.90	0.52	0.64	0.81	0.96	0.77	✓
	`omol`	2.60	1.04	1.27	0.93	0.50	0.67	0.85	0.00	✓
	UMA	2.60	1.22	1.46	1.02	0.71	0.88	0.97	0.00	✓
Coumarin	**MolCryst**	1.99	0.31	0.34	0.16	0.72	0.86	0.98	0.00	✓
	`omol`	1.99	1.27	1.43	1.13	0.72	0.87	0.39	0.06	✓
	Orb	1.99	8.00	8.50	2.75	−0.46	−0.57	−0.84	1.99	✓
	UMA	1.99	1.02	1.43	1.21	0.76	0.88	0.46	0.00	✓
Durene	**MolCryst**	1.50	0.94	1.15	1.01	−0.33	−0.50	−0.45	1.23	✓
	`omol`	1.50	0.90	1.11	0.96	0.33	0.50	0.26	1.23	✓
	UMA	1.50	0.92	1.13	1.01	−1.00	−1.00	−1.00	1.50	✓
Isonicotinamide	**MolCryst**	3.52	0.23	0.28	0.23	0.67	0.80	1.00	0.00	✓
	`omol`	3.52	4.95	5.71	3.37	0.00	−0.20	−1.00	3.28	×
	Orb	3.52	4.51	5.20	3.08	−0.33	−0.40	−1.00	3.28	×
	UMA	3.52	1.22	1.41	0.89	0.67	0.80	1.00	0.00	✓
Nicotinic acid	**MolCryst**	1.94	1.97	2.32	1.70	−0.33	−0.40	−0.74	0.76	✓
	`omol`	1.94	0.98	1.21	1.06	−0.67	−0.80	−0.11	0.87	✓
	Orb	1.94	0.95	1.18	1.02	−0.33	−0.40	−0.11	0.87	✓
	UMA	1.94	0.91	1.14	1.01	−0.33	−0.60	−0.02	0.87	✓
Niacinamide	**MolCryst**	5.42	0.64	0.95	1.14	0.78	0.92	0.87	0.00	✓
	`omol`	5.42	3.43	5.78	5.41	−0.39	−0.52	−0.79	5.42	×
	Orb	5.42	4.03	5.97	5.33	−0.59	−0.77	−0.90	5.42	×
	UMA	5.42	1.07	1.80	1.76	0.49	0.68	0.61	0.00	✓
Pyrazinamide	**MolCryst**	3.64	0.95	1.52	1.53	0.43	0.45	0.54	0.00	✓
	`omol`	3.64	2.19	2.62	2.72	−0.10	−0.20	−0.25	0.48	✓
	Orb	3.64	1.38	2.04	2.12	0.36	0.28	0.28	0.00	✓
	UMA	3.64	1.24	1.43	1.17	0.41	0.53	0.58	2.50	×
Resorcinol	**MolCryst**	5.64	1.68	1.96	1.37	0.67	0.80	0.91	1.86	✓
	`omol`	5.64	1.55	1.82	1.29	0.00	0.20	0.89	2.33	×
	Orb	5.64	1.23	2.21	2.43	0.00	0.20	0.31	0.00	✓
	UMA	5.64	2.05	2.50	2.07	0.00	0.20	0.89	2.33	×

Orb rows for durene and benzamide are omitted: only two durene polymorphs converged, for which rank correlations are undefined; three benzamide relaxations failed to reach the convergence criterion.

Table S9: Crystal polymorph data: density, lattice energy relative to the most stable form, and number of molecules in the unit cell. Bold entries indicate the most stable polymorph per system ($\Delta E_{\mathrm{latt}} = 0$). Green rows indicate polymorphs not included in the training set.

CSD Code	ρ (g cm^{-3})	ΔE_{latt} (kJ mol^{-1})	Z
BENZAC01	1.3293	2.98	4
BENZAC02	1.3261	1.84	4
BENZAC12	1.3877	0.00	4
BENZAC20	1.3730	0.45	4
BENZAC21	1.3683	0.57	4
BENZAC22	1.3240	1.86	4
BENZAC23	1.3107	2.18	4
BZAMID01	1.2831	0.00	4
BZAMID02	1.3348	1.84	4
BZAMID03	1.3213	1.21	4
BZAMID04	1.3213	1.21	4
BZAMID05	1.3104	0.78	4
BZAMID07	1.2723	2.18	4
BZAMID08	1.2785	0.84	4
BZAMID11	1.3296	2.60	4
BZAMID12	1.3077	1.66	4
BZAMID13	1.2622	3.41	32
BZAMID14	1.3081	0.68	4
BZAMID15	1.3086	0.74	4
BZAMID16	1.3172	2.07	4
BZAMID17	1.3283	2.57	4
BZAMID18	1.3096	0.77	4
BZAMID	1.3018	0.68	4

CSD Code	ρ (g cm^{-3})	ΔE_{latt} (kJ mol^{-1})	Z
COUMAR01	1.3996	1.47	4
COUMAR02	1.3988	1.47	4
COUMAR10	1.3993	1.52	4
COUMAR11	1.4334	1.81	4
COUMAR12	1.4003	1.46	4
COUMAR13	1.3611	0.06	2
COUMAR14	1.4066	3.06	8
COUMAR15	1.3557	2.56	8
COUMAR16	1.4240	3.36	12
COUMAR17	1.3731	2.75	12
COUMAR18	1.3898	1.99	4
COUMAR19	1.4163	1.52	4
COUMAR20	1.3819	0.00	2
COUMAR21	1.3973	1.44	4
COUMAR22	1.3983	1.44	4
COUMAR23	1.4246	1.63	4
DURENE01	1.0326	1.26	2
DURENE02	1.0357	1.94	2
DURENE03	1.0357	1.94	2
DURENE04	1.0357	1.94	2
DURENE05	1.0425	1.53	2
DURENE06	1.0865	0.03	2
DURENE07	1.0900	0.00	2
DURENE	1.0368	1.84	2

Table S8 – *continued*

CSD Code	ρ (g cm^{-3})	ΔE_{latt} (kJ mol^{-1})	Z
EHOWIH01	1.3919	3.48	4
EHOWIH02	1.3455	1.23	8
EHOWIH03	1.3522	2.46	8
EHOWIH04	1.3599	1.96	6
EHOWIH05	1.3452	0.00	4
EHOWIH06	1.3453	1.21	8
EHOWIH07	1.3733	3.28	4
EHOWIH	1.3936	3.52	4
NICOAC01	1.5182	0.87	4
NICOAC02	1.5180	0.83	4
NICOAC05	1.4990	1.94	4
NICOAC06	1.4838	2.54	4
NICOAC07	1.5326	0.00	4
NICOAC08	1.5204	0.76	4
NICOAC09	1.5261	0.37	4
NICOAM01	1.4421	0.47	4
NICOAM02	1.4274	0.59	4
NICOAM03	1.4274	0.59	4
NICOAM04	1.3515	3.18	16
NICOAM05	1.4263	0.61	4
NICOAM06	1.4392	0.00	4
NICOAM07	1.4449	2.82	2
NICOAM08	1.3984	1.71	4
NICOAM09	1.3921	2.88	40
NICOAM13	1.4380	0.47	4
NICOAM14	1.3603	3.34	16
NICOAM15	1.3925	2.98	16
NICOAM16	1.4241	1.96	8
NICOAM17	1.3732	5.89	4
NICOAM18	1.3803	3.96	4

CSD Code	ρ (g cm^{-3})	ΔE_{latt} (kJ mol^{-1})	Z
PYRZIN01	1.4135	0.00	4
PYRZIN02	1.3922	1.86	2
PYRZIN14	1.3685	0.48	4
PYRZIN15	1.4215	3.51	4
PYRZIN16	1.4349	2.45	2
PYRZIN17	1.1858	0.58	2
PYRZIN18	1.4442	0.83	4
PYRZIN20	1.2049	1.71	2
PYRZIN21	1.4126	2.94	4
PYRZIN22	1.4207	3.45	4
PYRZIN23	1.4422	0.78	4
PYRZIN25	1.4347	2.50	2
PYRZIN26	1.4347	2.39	2
PYRZIN27	1.4255	3.64	4
PYRZIN28	1.4151	2.94	4
PYRZIN29	1.4445	0.86	4
PYRZIN30	1.4405	0.73	4
PYRZIN	1.3805	1.85	4
RESORA03	1.3201	1.86	4
RESORA08	1.3391	5.64	4
RESORA09	1.3775	3.81	4
RESORA13	1.3113	2.33	4
RESORA15	1.3608	5.64	8
RESORA16	1.3645	0.00	4
RESORA24	1.3375	5.75	4
RESORA25	1.4050	2.90	4
RESORA27	1.3790	4.76	8
RESORA31	1.4671	4.73	8

C Structural Integrity for All Polymorphs

C.1 P2 Orientational Order Parameter

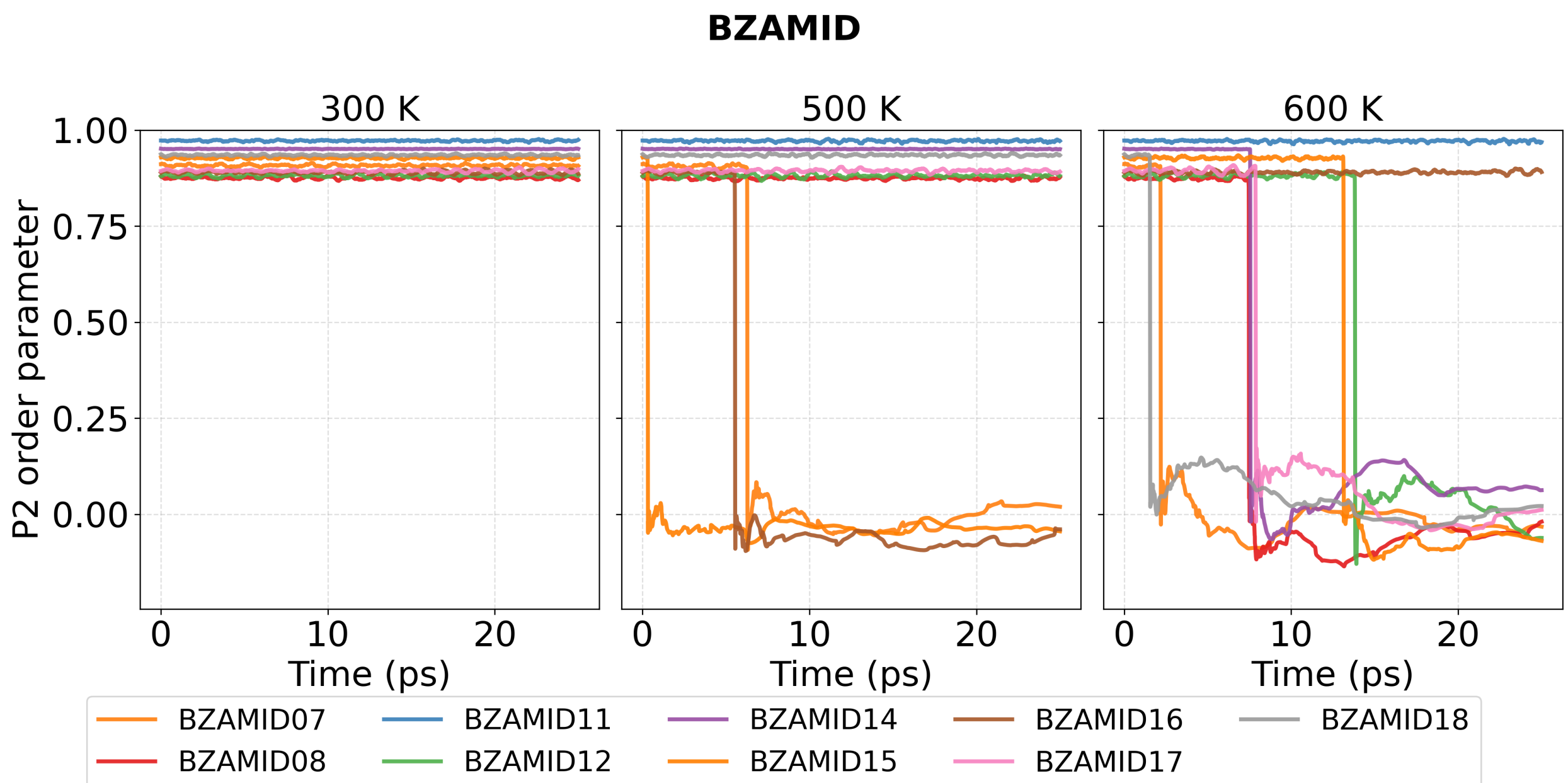

Figure S6: P2 orientational order parameter for all BZAMID polymorphs at 300, 500, and 600 K.

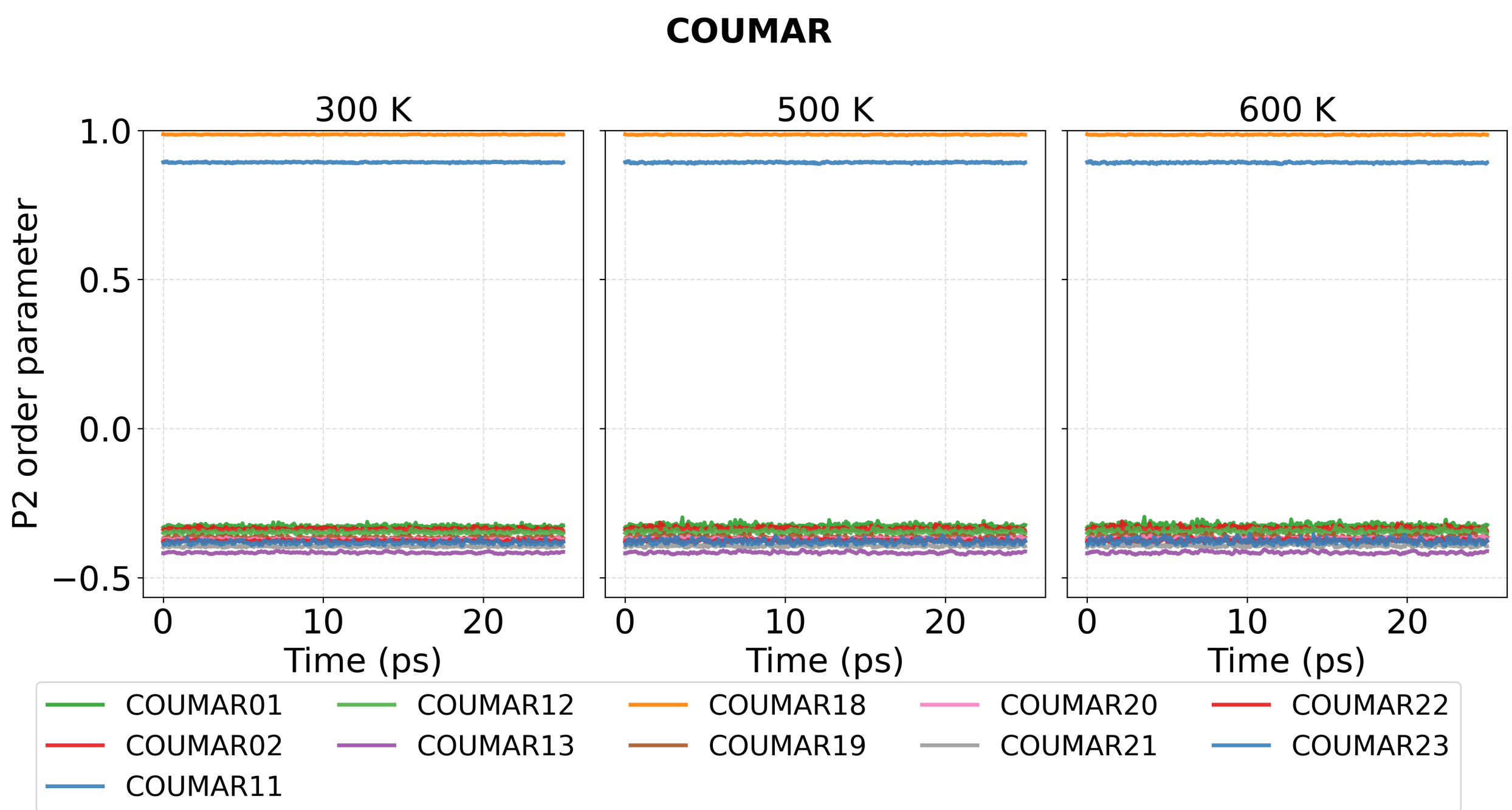

Figure S7: P2 orientational order parameter for all COUMAR polymorphs at 300, 500, and 600 K.

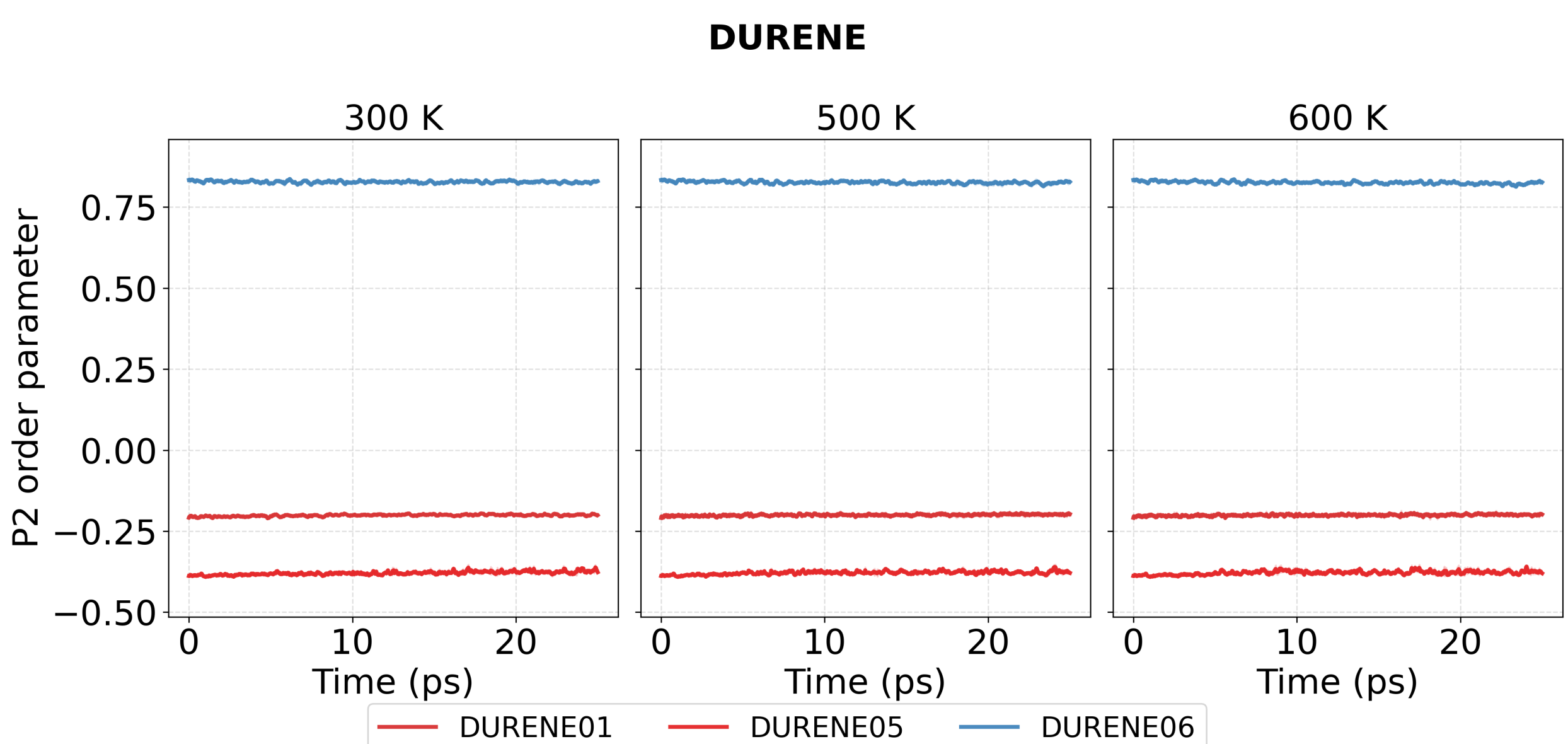

Figure S8: P2 orientational order parameter for all DURENE polymorphs at 300, 500, and 600 K.

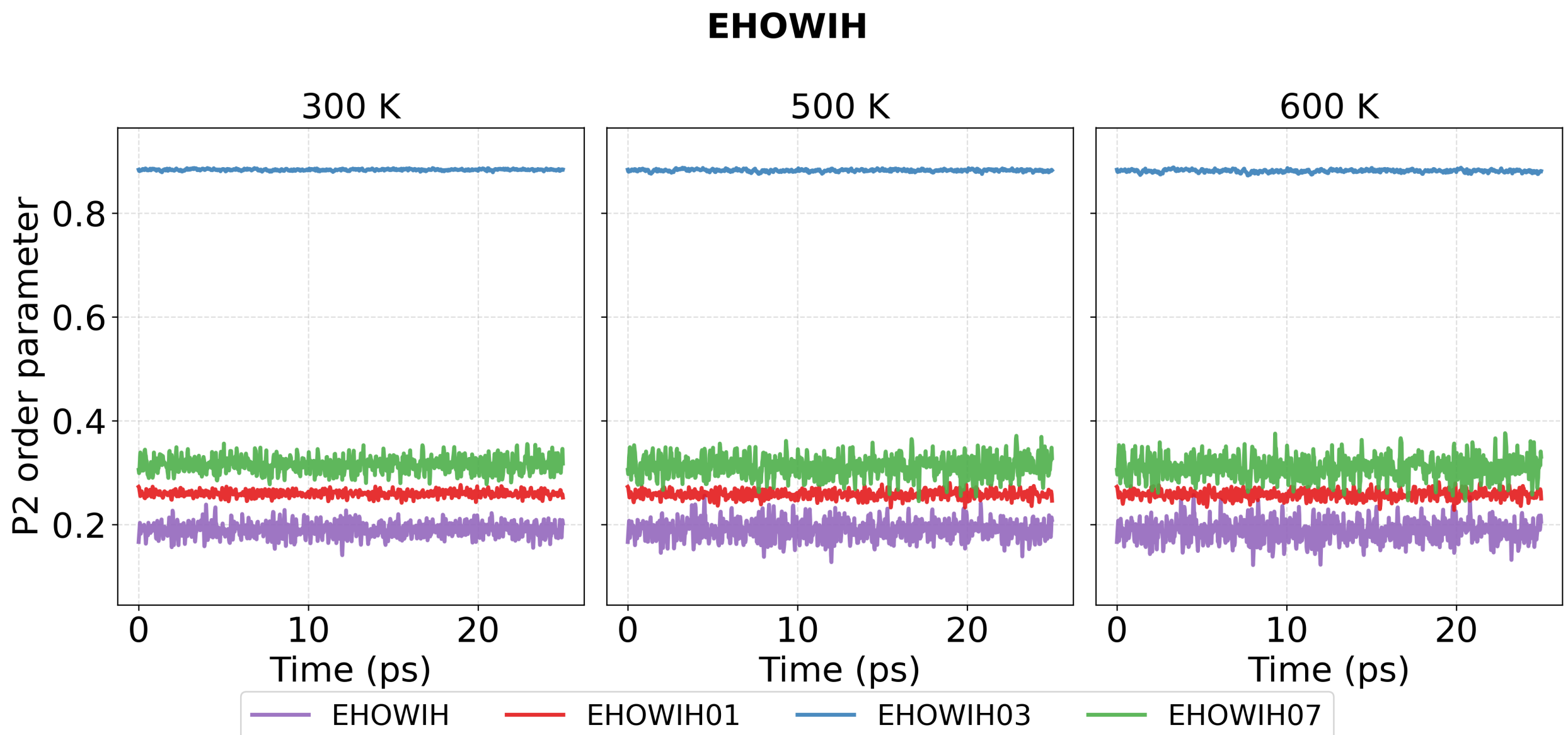

Figure S9: P2 orientational order parameter for all EHOWIH polymorphs at 300, 500, and 600 K.

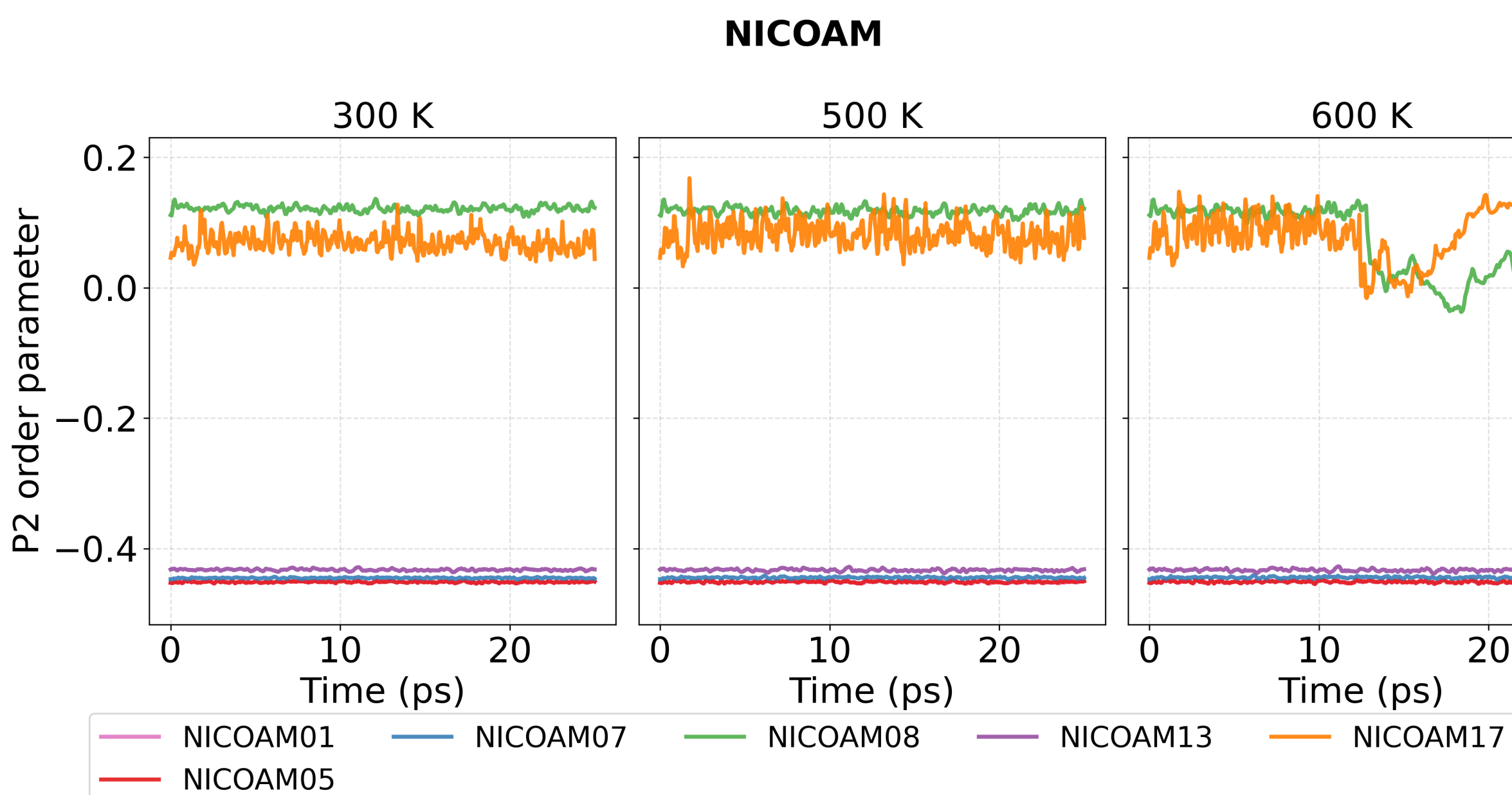

Figure S10: P2 orientational order parameter for all NICOAM polymorphs at 300, 500, and 600 K.

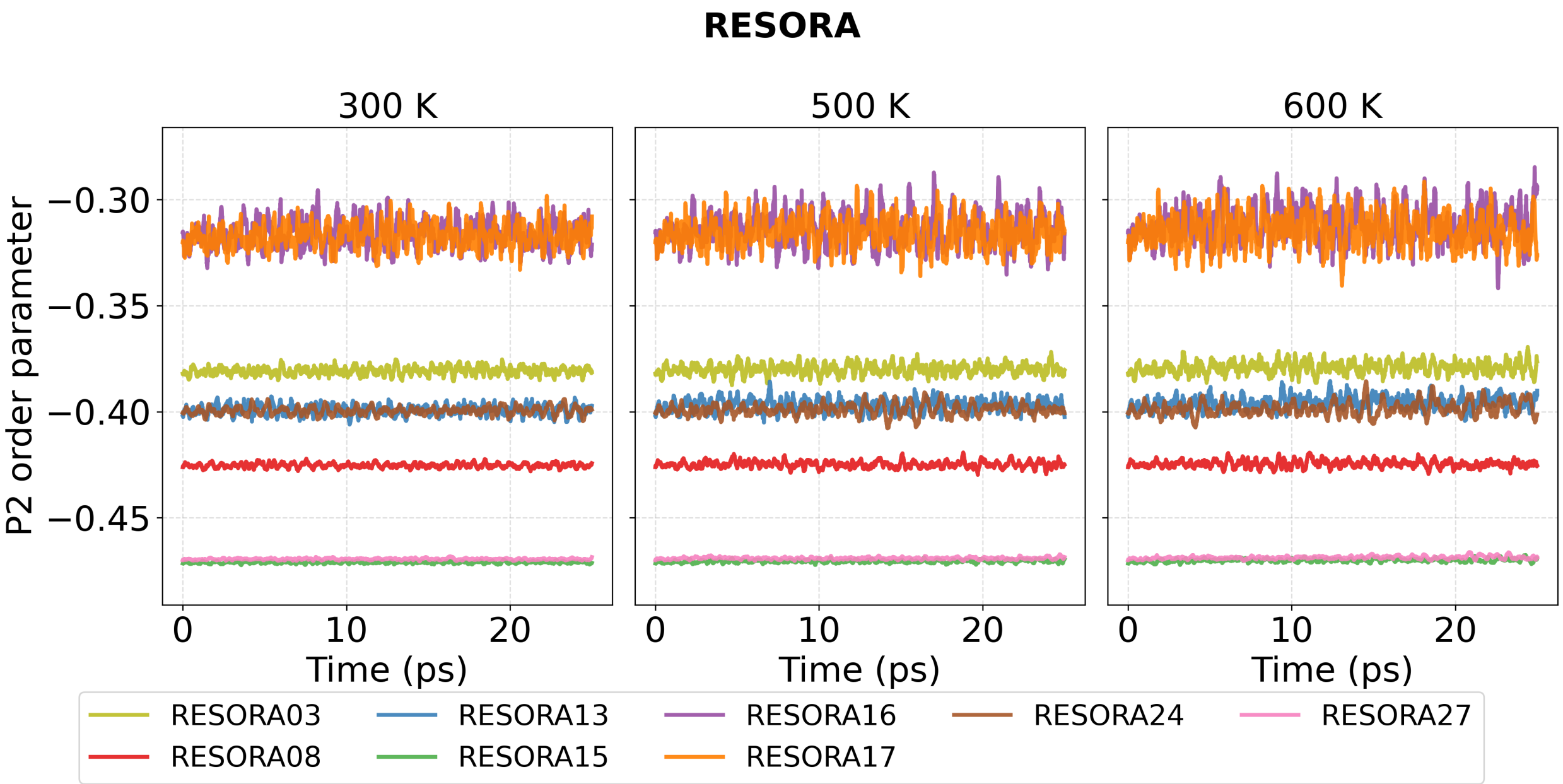

Figure S11: P2 orientational order parameter for all RESORA polymorphs at 300, 500, and 600 K.

C.2 Radial Distribution Functions

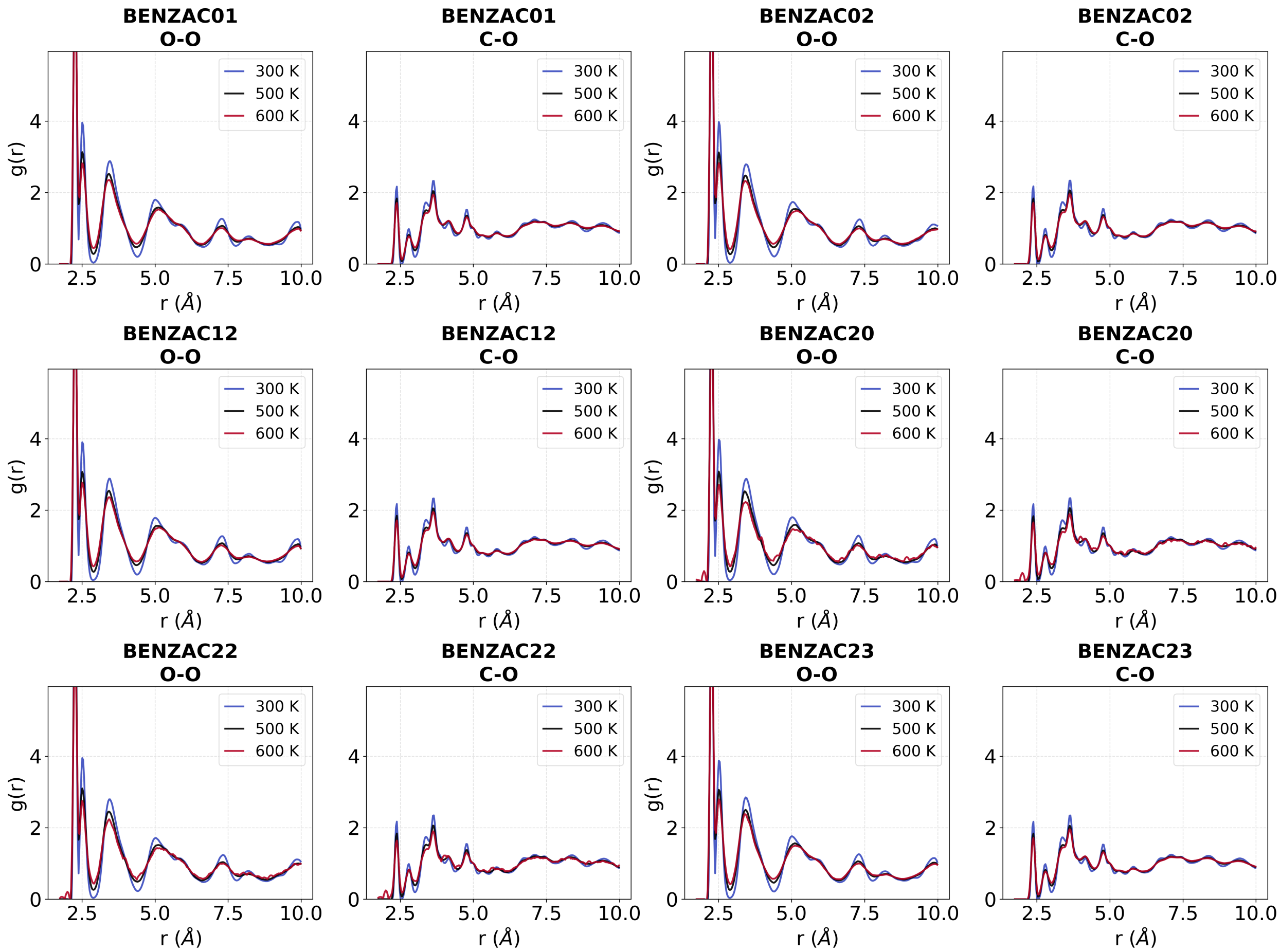

Figure S12: Radial distribution functions for all BENZAC polymorphs at 300, 500, and 600 K. Left panels show the intramolecular O–O pair; right panels show the intermolecular C–O pair.

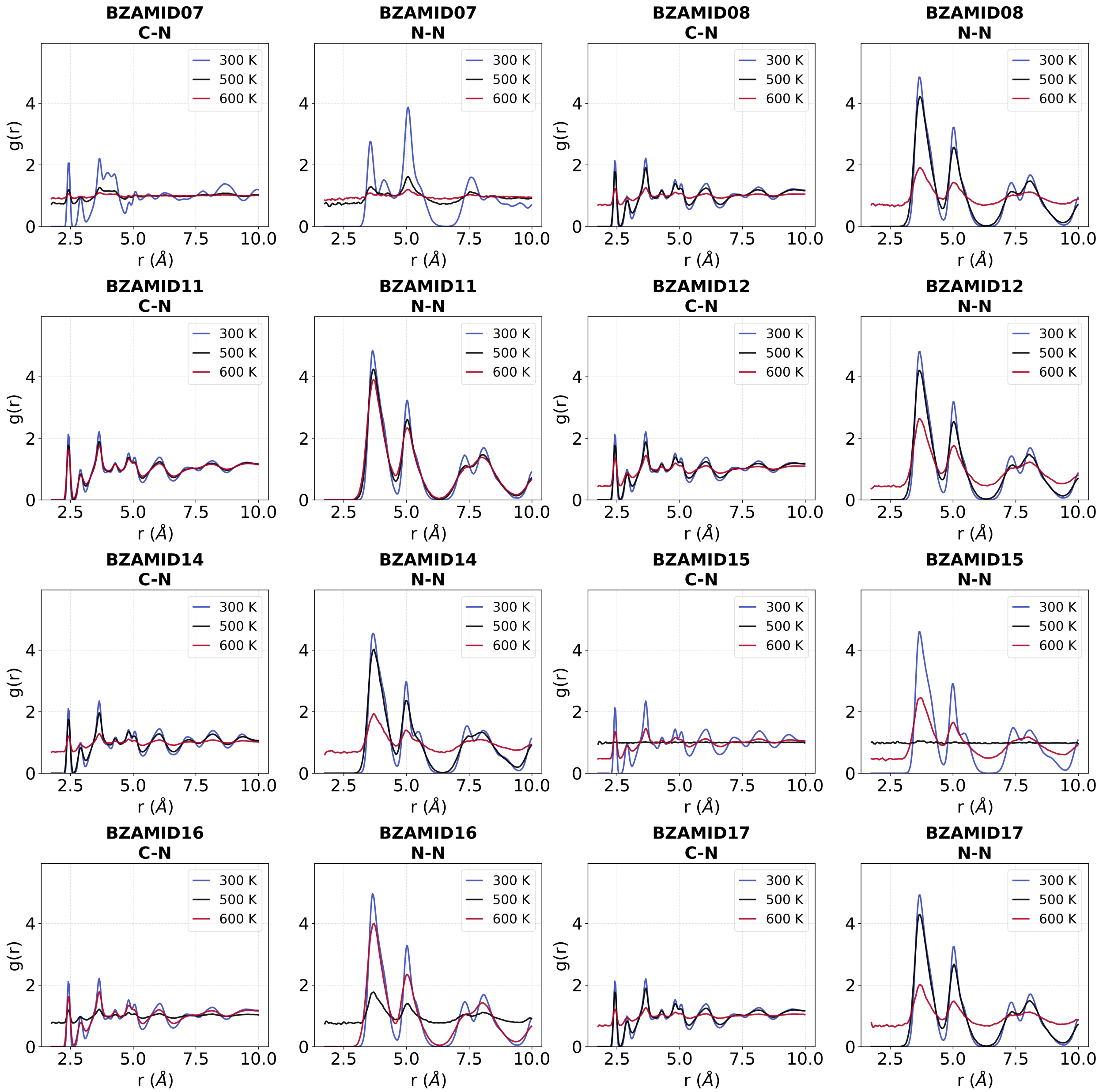

Figure S13: Radial distribution functions for BZAMID polymorphs (part 1) at 300, 500, and 600 K.

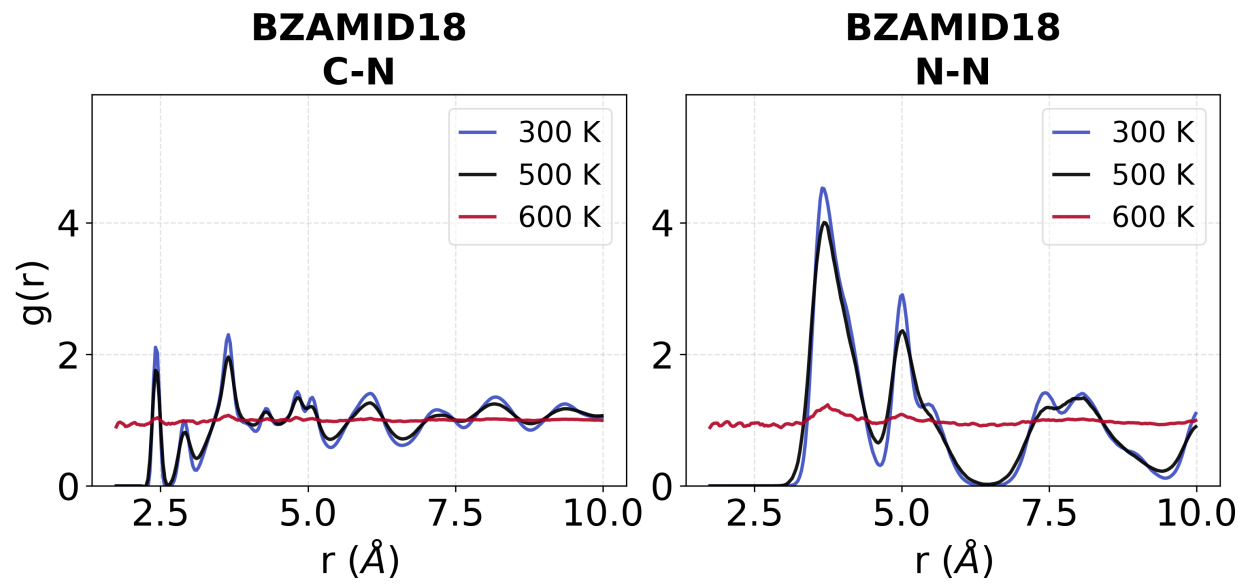

Figure S14: Radial distribution functions for BZAMID polymorphs (part 2) at 300, 500, and 600 K.

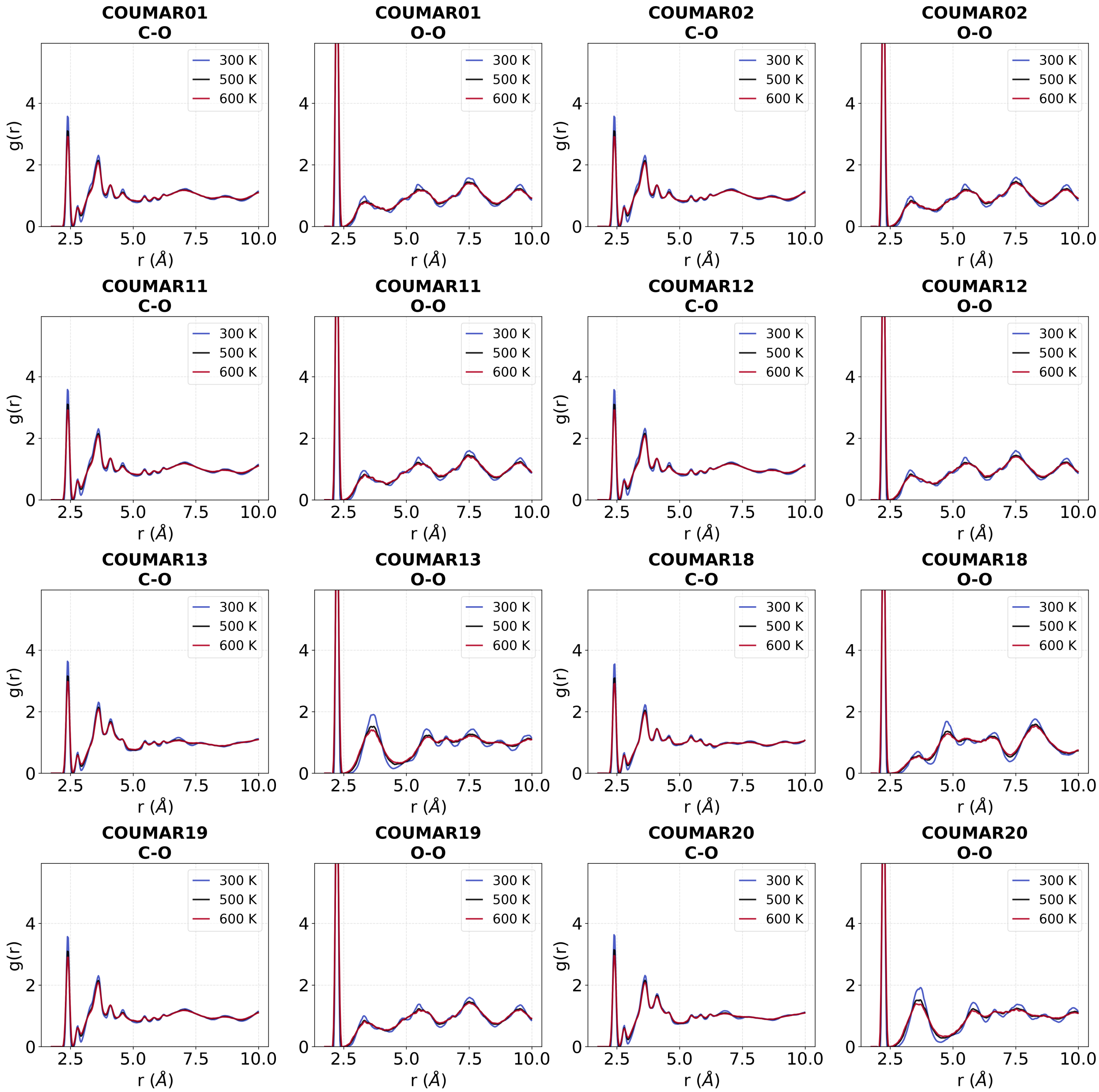

Figure S15: Radial distribution functions for COUMAR polymorphs (part 1) at 300, 500, and 600 K.

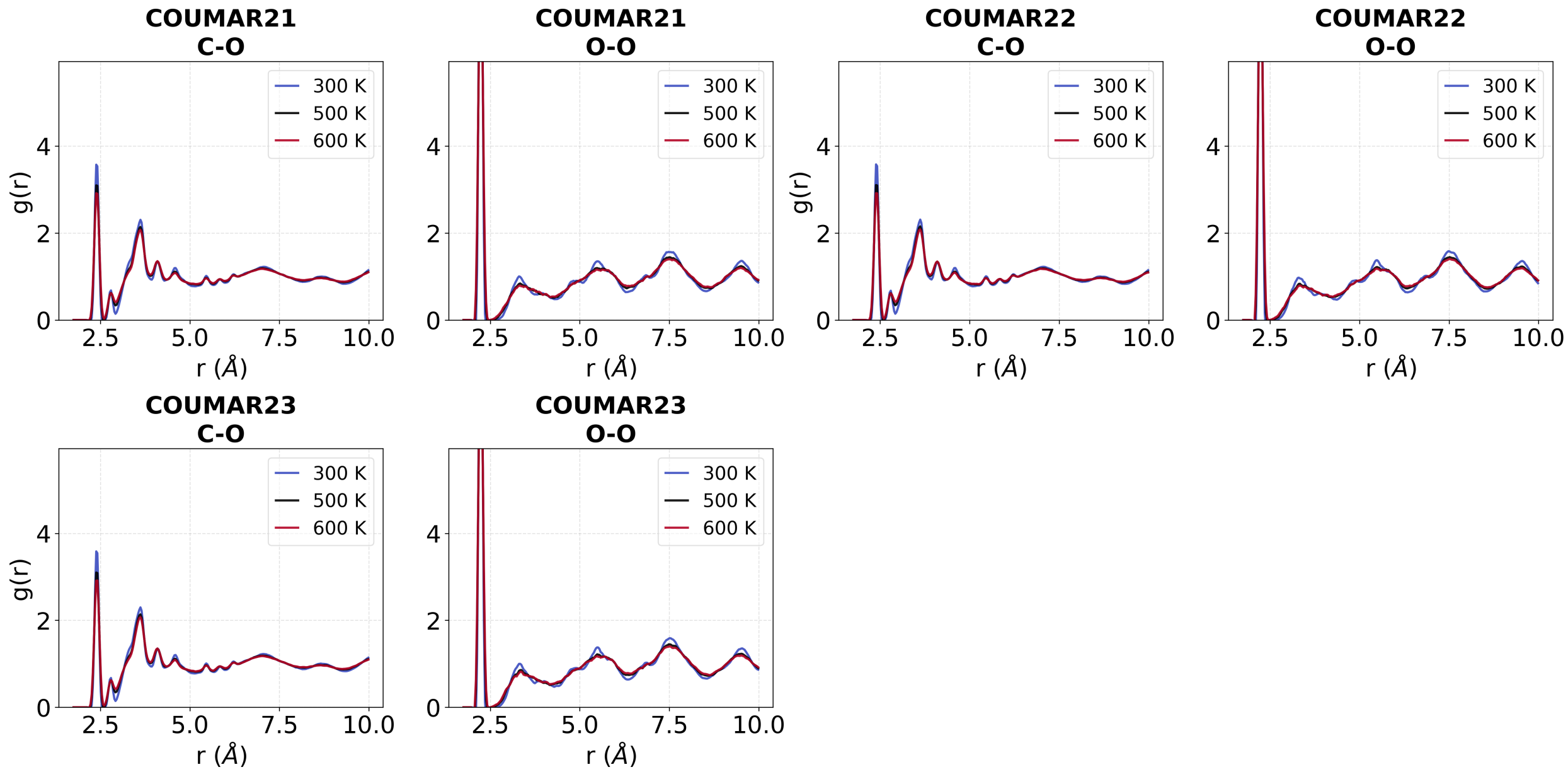

Figure S16: Radial distribution functions for COUMAR polymorphs (part 2) at 300, 500, and 600 K.

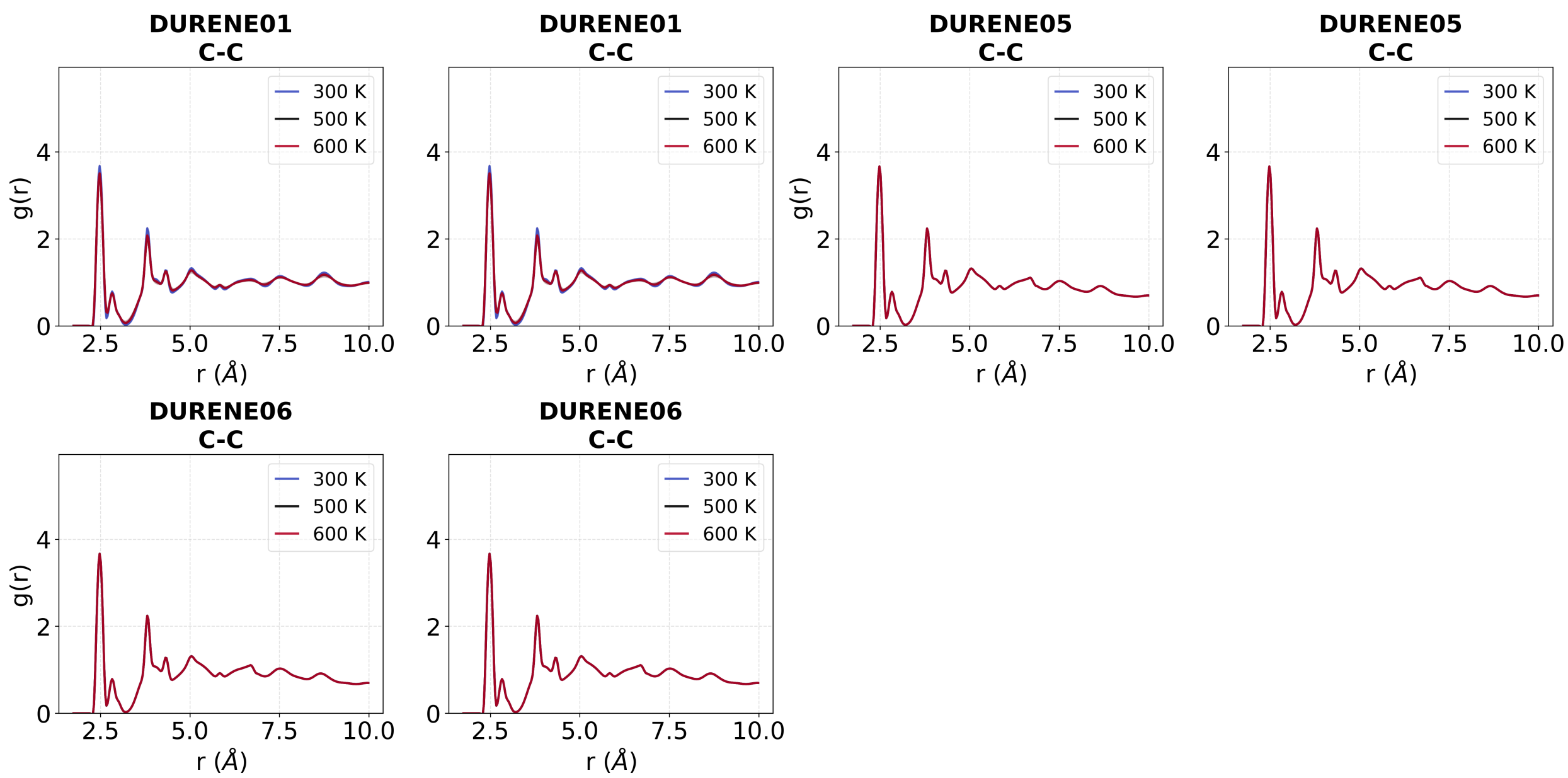

Figure S17: Radial distribution functions for all DURENE polymorphs at 300, 500, and 600 K.

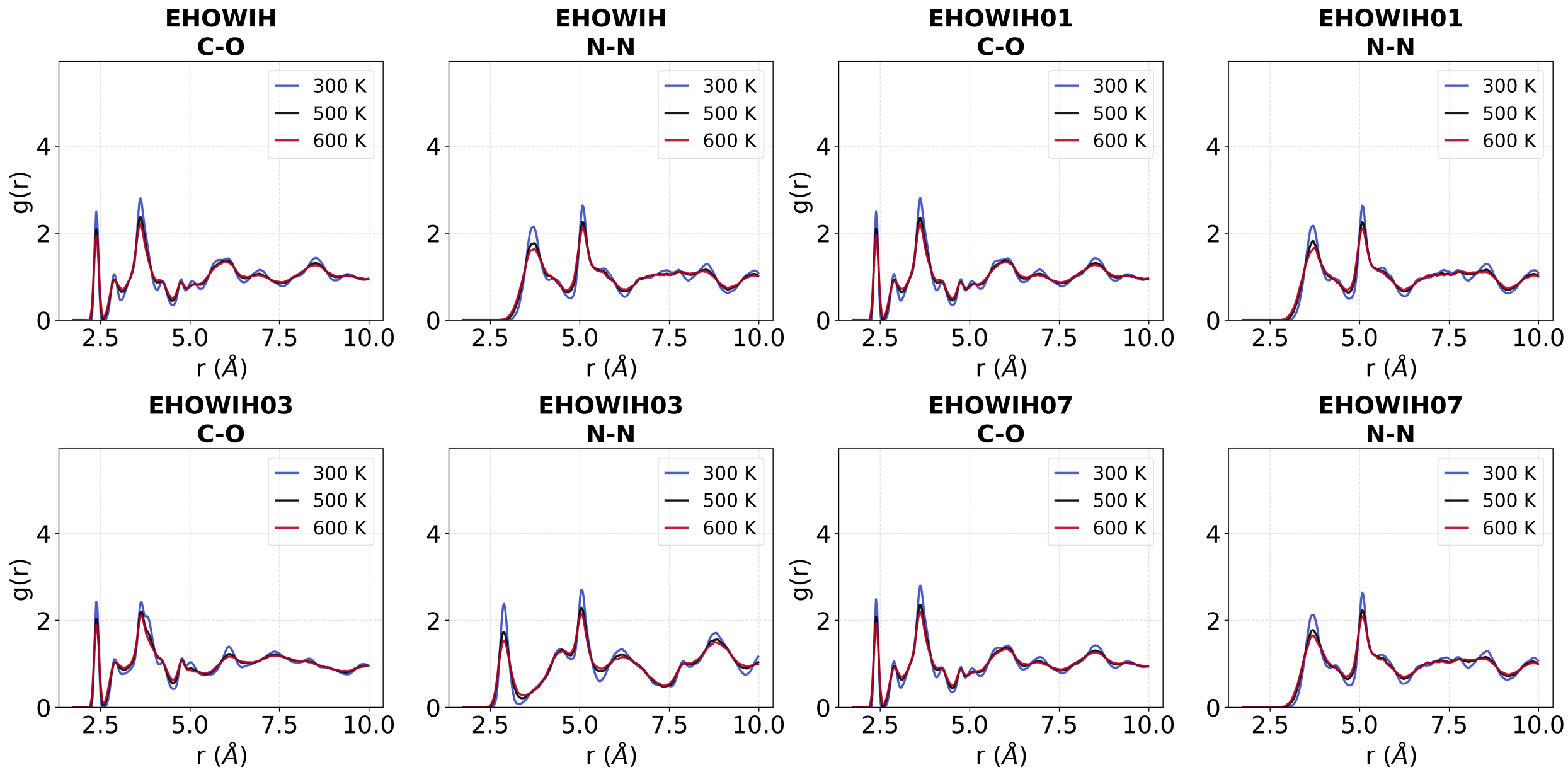

Figure S18: Radial distribution functions for all EHOWIH polymorphs at 300, 500, and 600 K.

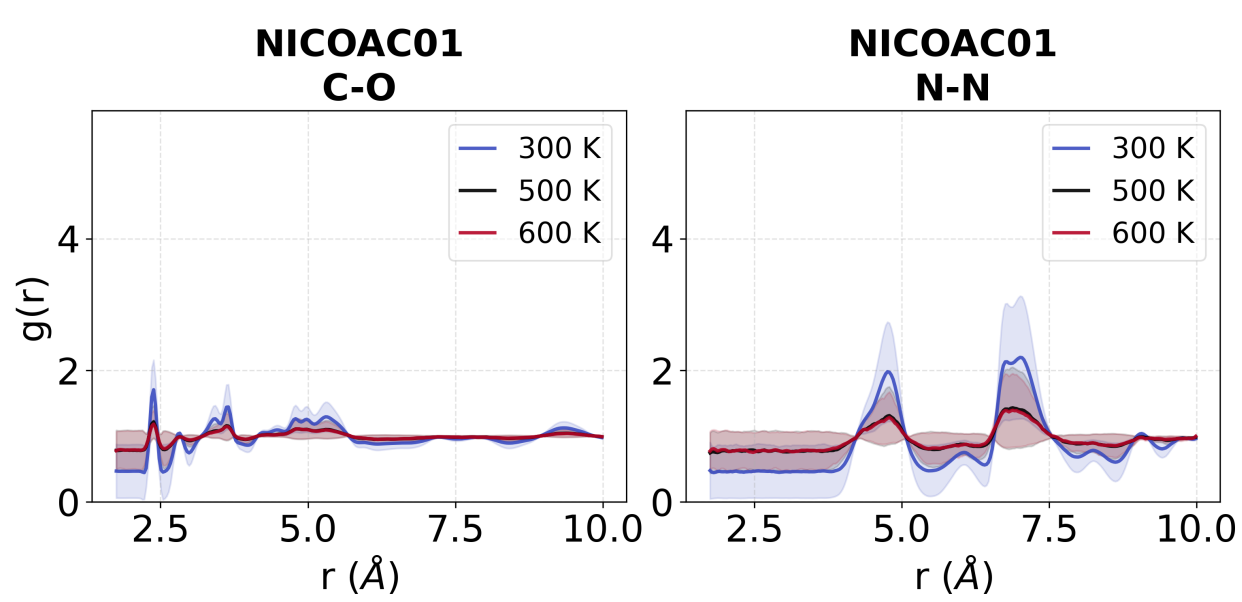

Figure S19: Radial distribution functions for all NICOAC polymorphs at 300, 500, and 600 K.

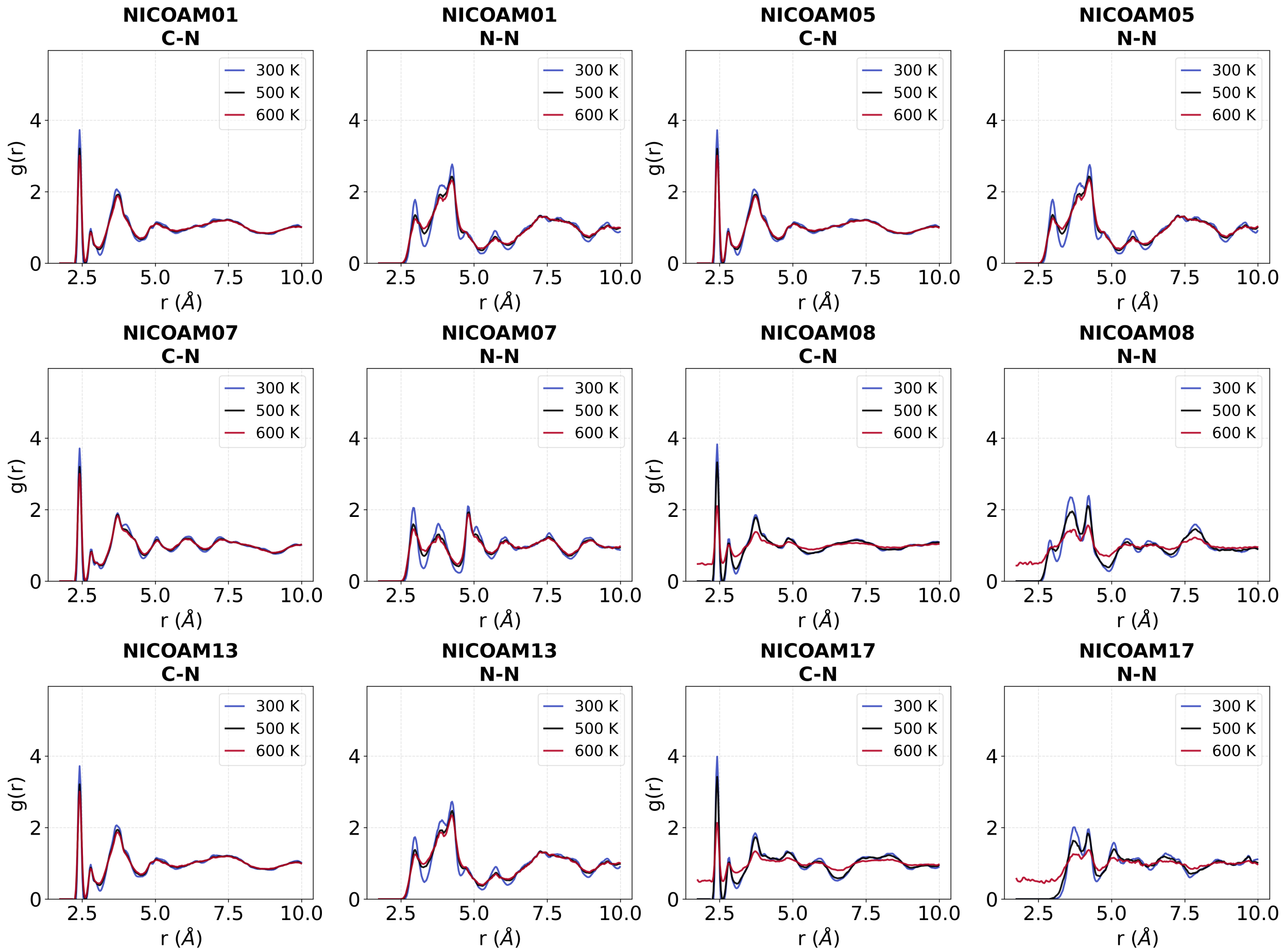

Figure S20: Radial distribution functions for all NICOAM polymorphs at 300, 500, and 600 K.

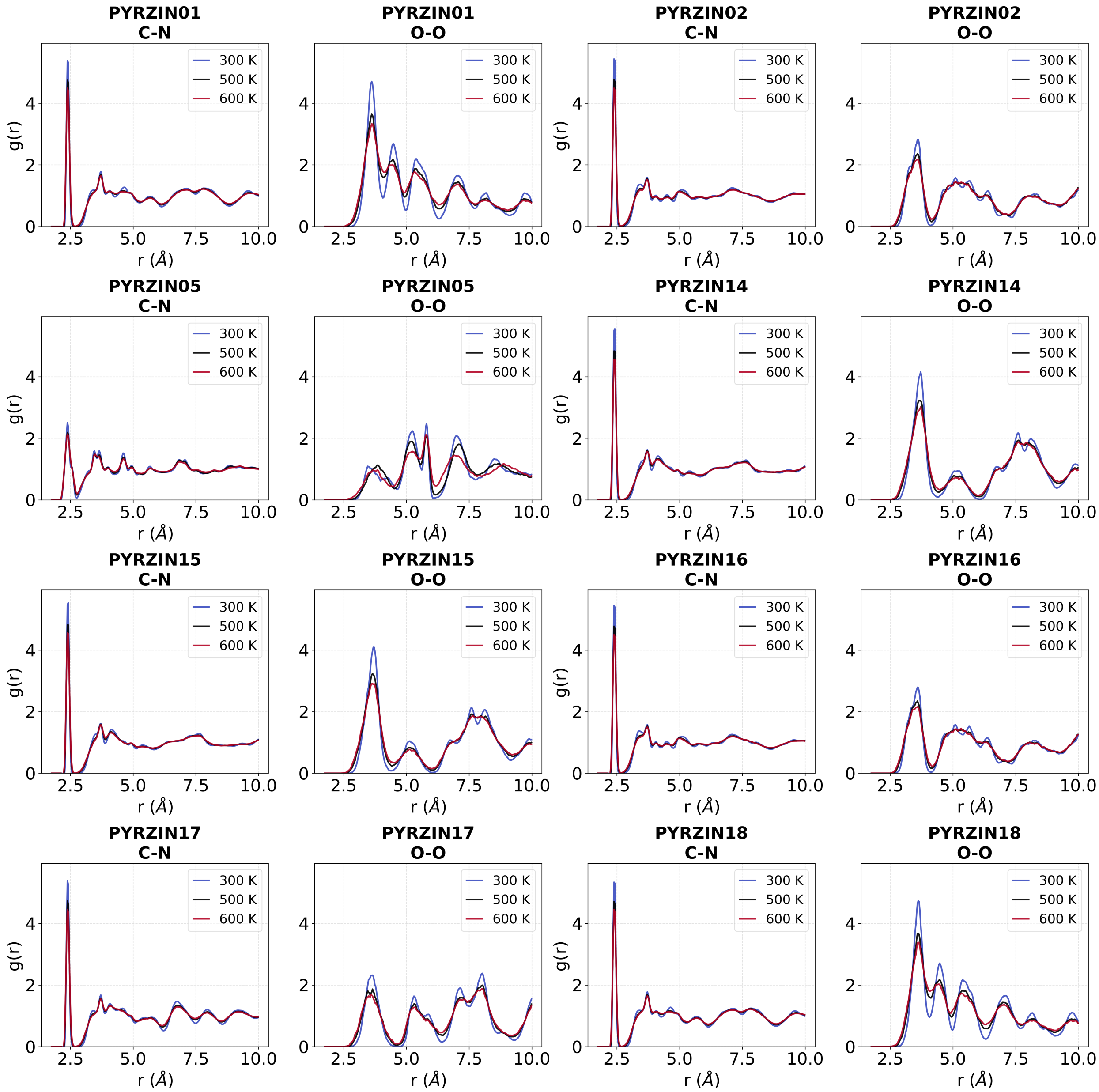

Figure S21: Radial distribution functions for PYRZIN polymorphs (part 1) at 300, 500, and 600 K.

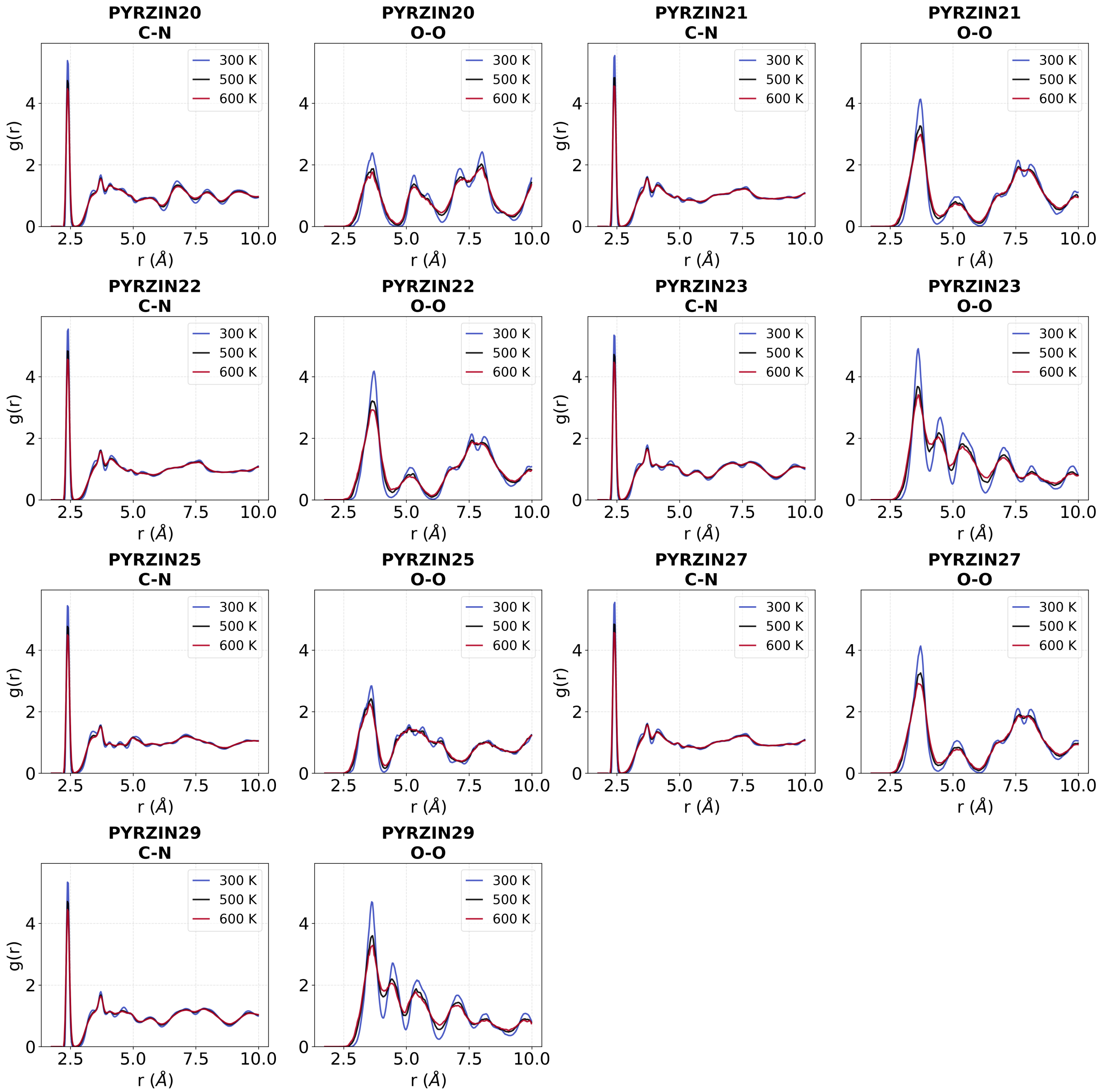

Figure S22: Radial distribution functions for PYRZIN polymorphs (part 2) at 300, 500, and 600 K.

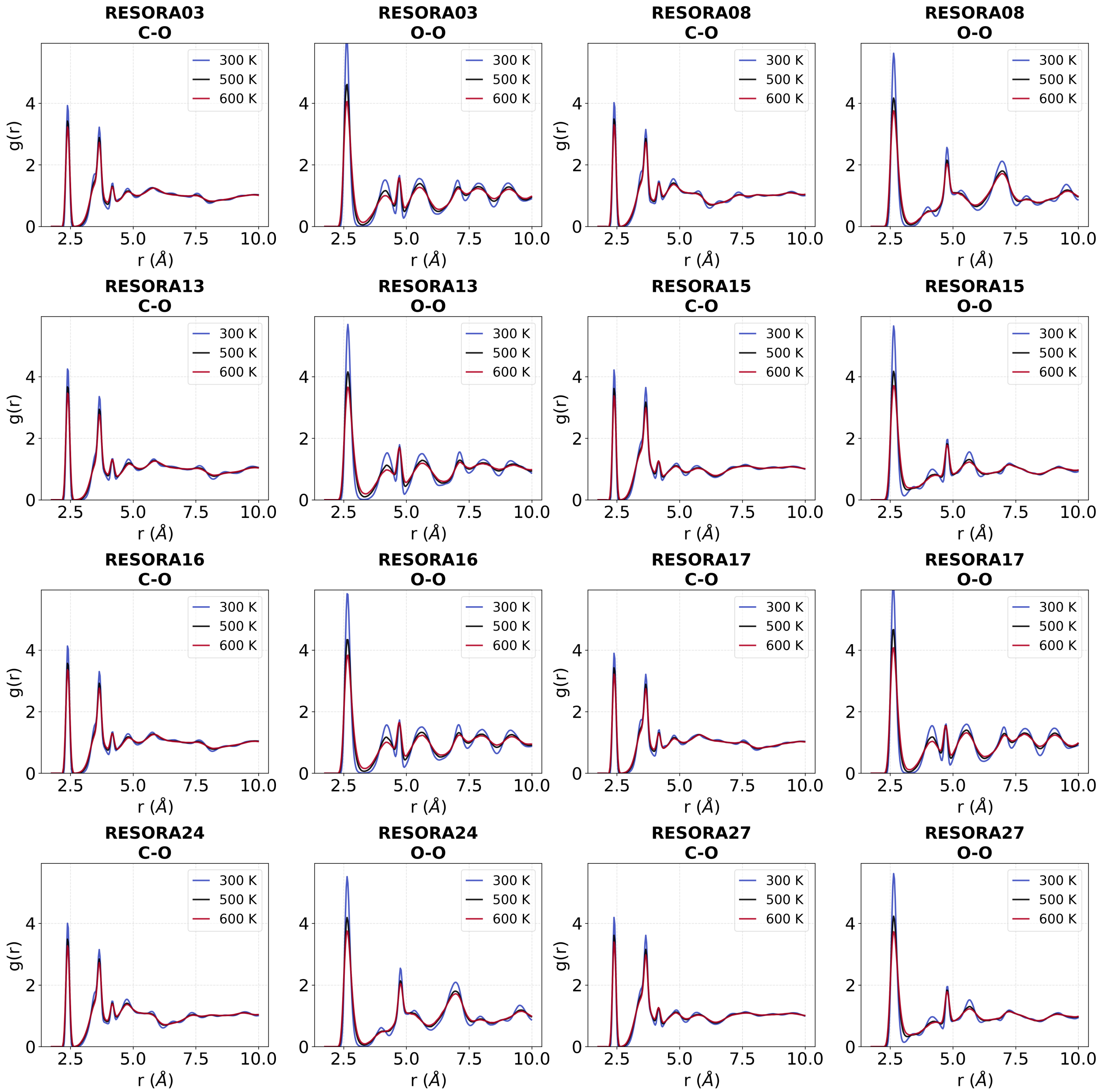

Figure S23: Radial distribution functions for all RESORA polymorphs at 300, 500, and 600 K.
